# Analyzing lignin biosynthesis pathways in rattan using improved co-expression networks of NACs and MYBs

**DOI:** 10.1186/s12870-022-03786-4

**Published:** 2022-08-24

**Authors:** Yu Wang, Yinguang Hou, Jiongliang Wang, Hansheng Zhao

**Affiliations:** 1 Institute of Gene Science and Industrialization for Bamboo and Rattan Resources, International Centre for Bamboo and Rattan, Beijing, 100102 China; 2 State Key Laboratory of Respiratory Disease, Guangzhou Institutes of Biomedicine and Health, Chinese Academy of Sciences, Huangpu District, Guangzhou, 510530 China

**Keywords:** *Calamus simplicifolius*, *Daemonorops jenkinsiana*, Gene co-expression network, *NAC* and *MYB* genes, Lignin biosynthesis pathway

## Abstract

**Background:**

The rattan is a valuable plant resource with multiple applications in tropical forests. *Calamus simplicifolius* and *Daemonorops jenkinsiana* are the two most representative rattan species, supplying over 95% of the raw materials for the rattan industry. Hence, the wood properties of both rattans have always attracted researchers’ attention.

**Results:**

We re-annotated the genomes, obtained 81 RNA-Seq datasets, and developed an improved pipeline to increase the reliability of co-expression networks of both rattans. Based on the data and pipeline, co-expression relationships were detected in 11 *NAC*s*,* 49 *MYB*s, and 86 lignin biosynthesis genes in *C. simplicifolius* and four *NAC*s, 59 *MYB*s, and 76 lignin biosynthesis genes in *D. jenkinsiana*, respectively. Among these co-expression pairs, several genes had a close relationship to the development of wood properties. Additionally, we detected the enzyme gene on the lignin biosynthesis pathway was regulated by either NAC or MYB, while *LACCASES* was regulated by both NAC and MYB. For *D. jenkinsiana*, the lignin biosynthesis regulatory network was characterized by positive regulation, and MYB possible negatively regulate non-expressed lignin biosynthesis genes in stem tissues. For *C. simplicifolius*, NAC may positively regulate highly expressed genes and negatively regulate non-expressed lignin biosynthesis genes in stem tissues. Furthermore, we established core regulatory networks of NAC and MYB for both rattans.

**Conclusions:**

This work improved the accuracy of rattan gene annotation by integrating an efficient co-expression network analysis pipeline, enhancing gene coverage and accuracy of the constructed network, and facilitating an understanding of co-expression relationships among NAC, MYB, and lignin biosynthesis genes in rattan and other plants.

**Supplementary Information:**

The online version contains supplementary material available at 10.1186/s12870-022-03786-4.

## Background

The rattan is the perennial evergreen climbing plant that belongs to the Calamoideae subfamily. Approximately 664 species are known globally, belonging to 13 genera, of which 27 commercial species are widely cultivated in tropical regions [1]. The rattan is one of the most widely used non-timber forest products after wood and bamboo [2]. Over the past five decades, the rattan furniture industry and international trade using rattan as a raw material have developed rapidly, forming a multi-billion-dollar global market, generating over 10 billion jobs, and playing a pivotal role in regional economic and social development [3]. Moreover, the rattan can be as beneficial to maintaining a natural ecosystem as complementary trees. They can be essential in replacing wood and protecting forest resources [4]. The rattan is a plant resource with multiple uses in tropical forests known as the “green gold” [3]. In addition to being nutrient-rich, rattan fruits and shoots are high-quality tropical fruits and forest vegetables. For example, the fruits of the genus *Daemonorops jenkinsiana* were used to prepare medicine known as the “Draconis Sanguis” [4]. Recent research has indicated that rattan was used to develop an advanced bone regeneration implant that can be used to treat extensively unloaded and weight-bearing bone injuries. It is expected that over 50,000 rattans are required each year for patient treatment [5]. Because the rattan plays a significant role in people’s production and daily lives, the rattan industry is referred to as the “sunrise industry of the 21st century”. Among all rattan species, *Calamus simplicifolius* and *Daemonorops jenkinsiana* are the two most critical rattan species, supplying over 95% of the raw materials for the rattan industry [6, 7]. However, the low use-value, insufficient production and supply of rattan, and the rigidity, fragility, and lack of elasticity of the cane limit the development of the rattan industry to some extent. Hence, the improvement of rattan properties is an essential contribution to resolving the low-quality development of the rattan industry. As a consequence, it has attracted the attention of researchers.

With the growing availability of RNA-Seq technologies, co-expression network analyses are increasingly utilized in data mining and analysis [8–10]. Constructing co-expression networks at different developmental stages or tissues makes it possible to decipher the regulatory network of specific developmental stages or tissues [10]. For example, Downs et al. studied different stages of maize development and constructed a developmental co-expression network, locating 24 modules associated with specific tissues and developmental stages [11–13]. Additionally, gene functions of non-model species are insufficiently studied compared to model species. Thus, co-expression network analysis facilitates transferring known functional annotations from model species to non-model species and identifying and refining gene functions for non-model species [14]. A co-expression network consisting of multiple species can also be used to identify conserved functional modules. For example, Ruprecht et al. discovered multiple conserved modules using co-expression networks across diverse plant species, specifically cell wall formation [15]. In addition to the above biological research applications, co-expression network was also utilized to develop plant molecular analysis platforms [16–19], providing researchers with tools for discovering gene functions. For example, Ma et al. developed a platform for co-expression network analysis on moso bamboo [19].

Modulus of elasticity (MOE) is essential for describing wood properties related to lignin content and composition [20]. Lignin is biosynthesized in higher plants through the phenylpropane biosynthesis pathway and the lignin biosynthesis-specific pathway, containing 12 gene families. The related regulation of the lignin biosynthesis pathway has been well studied in *Arabidopsis*, focusing on the *NAC* and *MYB* gene regulatory networks [21]. For example, two types of NAC domain transcription factors (NAM, ATAF1/2, CUC2) have been demonstrated to act as transcriptional switch factors to regulate the composition of vascular tissue duct cells and secondary fibroblast walls, respectively, in *Arabidopsis*. Studies have shown that NAC-like transcription factors in poplars, rice, maize, *Medicago sativa*, and other species display similar secondary wall synthesis switch functions in *Arabidopsis* [22–24]. NAC-like transcriptional factors have been demonstrated in all vascular plant groups, from lower vascular plants to angiosperms. Still, the presence of these genes in rattan has not been reported. MYB family transcription factors are critical secondary regulators of secondary wall biosynthesis. Most of them are downstream of the NAC transcription switch [25], e.g., MYB46 and MYB83 are directly controlled by NAC transcription factors. The studies found that the MYB46 and MYB83 double mutants produced more severe secondary wall synthesis defects than the nst1nst3 double mutants. The overexpression of the MYB46 and MYB83 transcription factors may result in the up-regulation of genes that encode different secondary wall components, including cellulose, xylem, and lignin [26]. These findings indicated that MYB46 and MYB83 play an imperative role in secondary wall deposition in fibroblasts and tubular cells as secondary regulators regulated by NAC transcription factors [27–29]. Further, the homologous genes *PtMYB4*, *EgMYB2*, *PtrMYB3*, and *PtrMYB20* of MYB46 and MYB83 have also been found in woody plants, such as pine, *Eucalyptus grandis*, and poplar trees, which can also activate secondary wall biosynthesis-related genes [25]. However, there are few reports on the MYB transcription factor in rattan.

In the *NAC* and *MYB* gene regulatory networks, the VASCULAR-RELATED NAC-DOMAIN 1–7 (VND1–7), the NAC SECONDARY WALL THICKENING PROMOTING FACTOR 1–3 (NST1–3), and the SECONDARY WALL-ASSOCIATED NAC DOMAIN PROTEIN 1 (SND1) are the first-layer main switches. MYB46 and MYB83 are downstream targets of NAC proteins, both of which act as second-layer master switches that influence downstream genes involved in the secondary wall formation [21, 30]. MYB58 and MYB63 are regulated by homologs of MYB46 and SND1 (NST1, NST2, VND6, and VND7) [31]. These proteins directly activate downstream genes (i.e., *PAL*, *C4H*, *4CL*) involved in the lignin biosynthesis pathway [32]. In addition, MYB58, MYB63, and MYB85 are specific transcription factors for the lignin biosynthesis pathway since their overexpression causes ectopically deposition of lignin [33, 34]. Besides *Arabidopsis*, some transcription factors involved in the lignin biosynthesis pathway are also found in some trees. For instance, MYB1 [35] and MYB4 [36] in *Pinus taeda* L. and MYB2 [37] in *Eucalyptus grandis* can bind to the AC element in the promoter region of the lignin biosynthesis genes and have been shown to regulate the pathway. However, few studies have explored NAC and MYB in regulating lignin biosynthesis pathways in the rattan [38].

Here, we sequenced the transcriptome of 81 samples with different developmental stages and rattan tissues, updated their genome annotation, and developed a pipeline to construct co-expression networks. Based on the above data and methods, the regulatory mechanisms of NAC and MYB transcription factors were investigated in the rattan’s lignin biosynthesis pathway. Thus, this study aims to provide new insight into gene annotations and co-expression networks as well as identify and describe key regulatory networks associated with NAC and MYB.

## Results

### Genome annotation update and evaluation

To update the genome annotation of *C. simplicifolius* and *D. jenkinsiana*, we identified their repetitive sequences first. According to the results (Additional file 1: Table S1), there were 2,140,483 (accounting for 62.48% of the genome) and 1,864,083 (57.38%) repeat sequences; there were 361,789 (36.86% of the genome) and 242,572 (30.11%) of long terminal repeat (LTR) retrotransposons in *C. simplicifolius* and *D. jenkinsiana*, respectively. As compared to other genomes, the proportion of long terminal repeat-retrotransposons (LTR-RTs) in both rattans was lower than 70.1% of maize genome [39], 54.97% of moso bamboo genome and 55% of sorghum genome [40], but higher than 26% of rice genome [41]. Ty1-copia and Typ3-gypsy were two main superfamilies of LTR-RTs. In *C. simplicifolius*, 205,501 (19.71% of the genome) and 149,525 (16.93%) were identified, while 116,899 (17.62% of the genome) and 121,625 (12.26%) were obtained in *D. jenkinsiana*. The analysis of the ratio of Ty3-gypsy to Ty1-copia revealed the ratios of *C. simplicifolius* and *D. jenkinsiana* to be 7.3:10 and 9.6:10, respectively, which were lower than those of moso bamboo (13:10) [40], soybean (14:10) [42], maize (16:10) [39], and far lower than rice (49:10) [43] and sorghum (37:10) [44].

After masking repetitive sequences in the genomes, we performed the genome structure annotations using three methods, i.e., homolog alignment, RNA-Seq data alignment, and ab initio prediction. To perform the homolog prediction, we selected the protein sequences of seven closed species based on the relationship between rattan and other species. These species included *Arabidopsis*, *Brachypodium*, foxtail millet, maize, oil palm, rice, and sorghum. After removing repetitive sequences from 347,477 sequences, we obtained 313,341 sequences used as inputs for homolog prediction. The homolog method revealed that 75,682 and 73,653 gene models were predicted for *C. simplicifolius* and *D. jenkinsiana*, respectively. Additionally, for RNA-Seq data prediction, we analyzed 81 transcriptome datasets in the gene prediction analysis of RNA-Seq data, including 35 datasets for *C. simplicifolius* and 46 datasets for *D. jenkinsiana* (Additional file 1: Table S2 and S8). After quality control, 62,370 gene models were predicted for *C. simplicifolius*, and 61,939 were predicted for *D. jenkinsiana*, including the completed gene models were 34,619 and 34,005. Gene models lacking the 3′ UTR were 13,188 and 13,124, those lacking the 5′ UTR were 5511 and 5512, and those lacking both ends were 9052 and 9298. In addition, ab initio prediction integrated the homolog and transcriptome prediction results. Redundant gene models with high similarity were removed. Thus 32,041 and 31,532 genes were obtained in *C. simplicifolius* and *D. jenkinsiana*, respectively. We selected 3698 optimal gene models of *C. simplicifolius* and 3684 of *D. jenkinsiana*. By running the first training, we obtained 74,539 gene models of *C. simplicifolius* and 78,379 of *D. jenkinsiana*. As a result of running the second training, there are 86,595 gene models of *C. simplicifolius*. However, the accuracy of HMM was not improved in *D. jenkinsiana*, so the first training result of *D. jenkinsiana* was used. Gene numbers of the gene models obtained by an ab initio prediction method were 86,595 and 78,379 in *C. simplicifolius* and *D. jenkinsiana*, respectively. Using AUGUSTUS to predict gene models, the accuracy was 63.3 and 64.54%, respectively. In sum, we integrated the above three predictions and constructed complete gene models of 51,034 of *C. simplicifolius* and 50,108 of *D. jenkinsiana*.

### Evaluation and functions of genome annotation

**Fig. 1:**
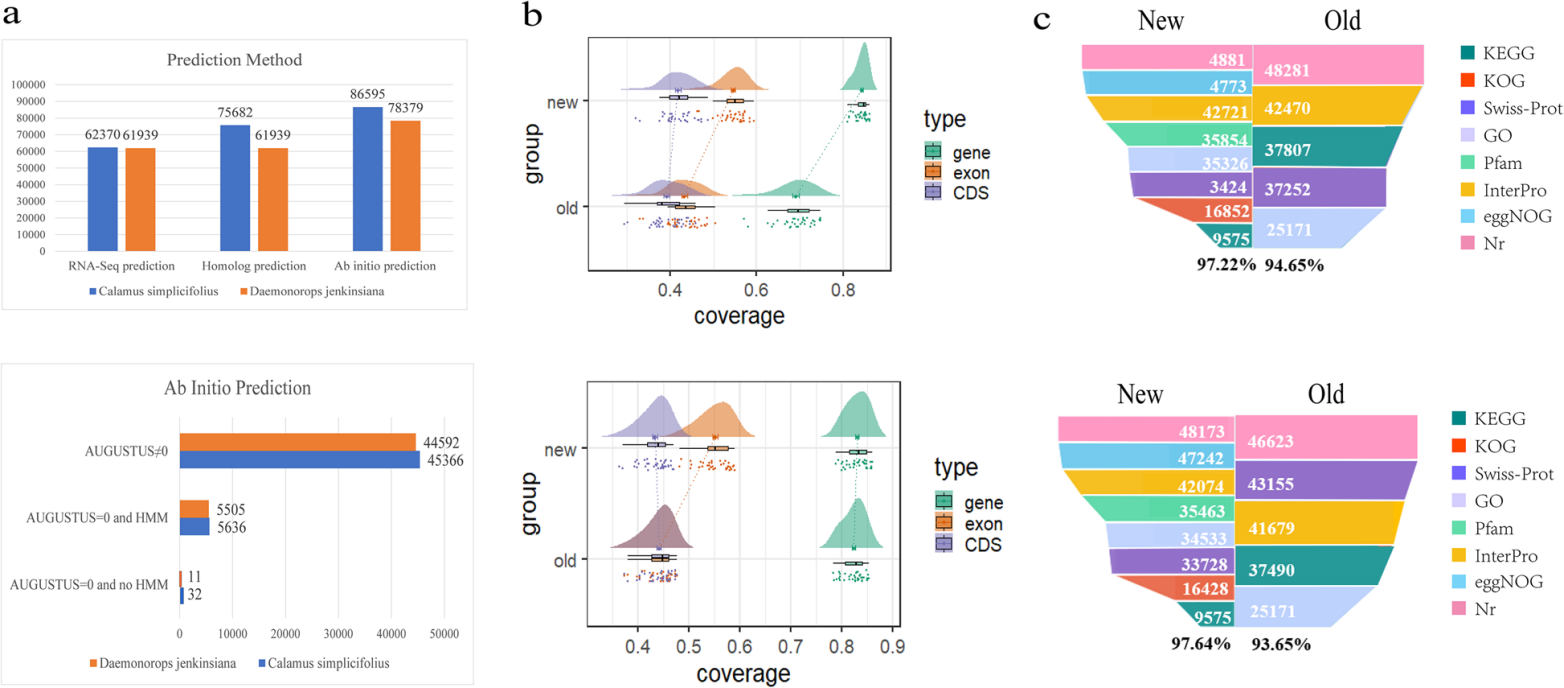
Evaluation of gene annotation of *C. simplicifolius* and *D. jenkinsiana*. **a** Number of genes obtained by three gene prediction methods **b** Alignment of transcriptome data with different annotations. **c** Comparison of annotations of gene functions (the top is *C. simplicifolius* and the bottom is *D. jenkinsiana*)

We evaluated gene annotation results from three perspectives. The first aspect was the evaluation of gene prediction (Fig. 1a). In *C. simplicifolius* and *D. jenkinsiana*, 45,366 and 44,592 genes were supported by RNA-Seq and homolog data, respectively, and 5668 and 5516 genes were ab initio predicted. HMM models do not support only 32 and 11 genes. Thus, 99.94% (51,002 genes) and 99.98% (50,997 genes) were validated by evidence for *C. simplicifolius* and *D. jenkinsiana*, respectively, implying that the annotation results were reliable. Additionally, the predicted gene models were evaluated using the BUSCO software for completeness. BUSCO assessment (Additional file 1: Table S3) showed that the genome sequence level was 80.10 and 80.70% in *C. simplicifolius* and *D. jenkinsiana*, respectively. Compared to the previous version of *C. simplicifolius*, the number of annotated genes was decreased by 201. However, the number of exons was increased by 55,268, and the number of CDS was increased by 55,724. The number of annotated genes in the updated version of *D. jenkinsiana* was decreased by 3234 genes. In contrast, the number of exons and CDS increased by 15,875 and 6868, respectively. Hence, some incorrect annotations were removed and more accurate gene models were identified in the updated version. Thus, BUSCO demonstrated that 70.6 and 72.5% of the conserved proteins were present in *C. simplicifolius* and *D. jenkinsiana*, respectively.

The gene structure was observed by comparing it to the previously published and updated annotations across 81 transcriptome datasets (Additional file 1: Table S2). The raincloud plots provided an overview of the number of bases overlaid in the gene, CDS, and exon region. According to the results (Fig. 1b), the updated annotation improved coverage of the mean point on the core density map for *C. simplicifolius* at the exon, CDS, and gene level. This was in line with IGV v2.5.3 (Additional file 2: Fig. S1a); the latest annotation version increased the number of exons and corrected the site of the previous gene error. The gene-level core density plot has higher peaks than in the previous version. Accordingly, the average coverage of the annotated genes in the updated version of the 35 samples data was relatively high. This demonstrated that the annotation of the latest version of *C. simplicifolius* was more accurate. In addition, the raincloud plots of *D. jenkinsiana* illustrated that the coverage at the exon level was improved in the updated annotation version. In contrast, the CDS and gene-level coverage were not improved substantially. This was also consistent with IGV results (Additional file 2: Fig. S1b). The locus information in the two versions was essentially the same. In addition, the CDS region and the exon sequence overlapped in the previous version. The CDS and exon had the same locus information in the previous annotations, indicating that the results were not rigorous enough.

Additionally, we performed a genome-wide functional annotation (Fig. 1c). Thus, 49,619 genes (97.22%) and 48,929 genes (97.64%) were annotated in *C. simplicifolius* and *D. jenkinsiana*, respectively. Five database annotations were used in the previous version, but eight were used in the updated version. Also, the number of annotations in the Nr, InterPro, and GO databases had increased in the updated version, while the number of annotations in the KEGG and Swiss-Prot databases had decreased. The number of annotations in the updated version of the KEGG differed substantially from that of the previous version. This may be affected by the different versions of the KASS database we used.

### Construction of rattan co-expression networks using an integrated pipeline

We improved a co-expression network construction process to retrieve more meaningful gene pairs and enhance credibility. The process was then applied to the analysis of both rattans. The first step was to construct a co-expression network using the WGCNA method. Outlier samples were undetected in phylogenetic analysis (Additional file 2: Fig. S2). Therefore, all RNA-Seqs were used for subsequent correlation analysis. The soft threshold (Additional file 2: Fig. S2) showed the adjacency matrix constructed by both rattans. The optimal soft thresholds for *C. simplicifolius* and *D. jenkinsiana* were 7 and 9, respectively, and gene pairs that meet the TOM > 0.1 requirement were selected. We thus identified 98,093,582 and 18,498,588 gene pairs in *C. simplicifolius* and *D. jenkinsiana*, respectively.

**Fig. 2:**
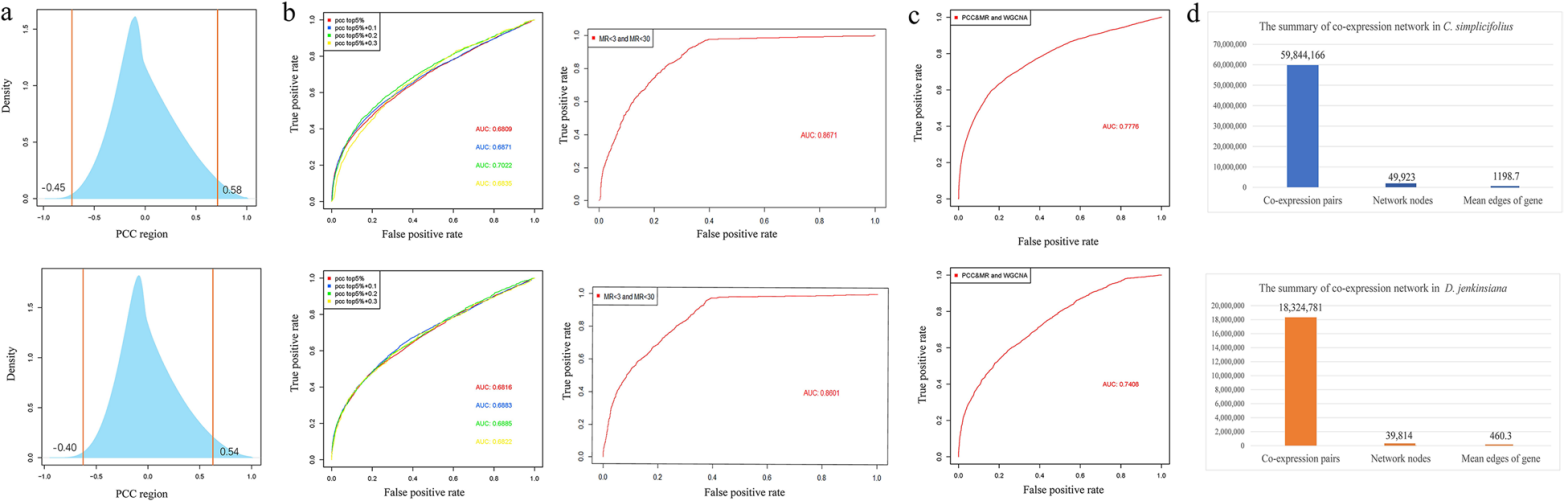
Construction and evaluation of the rattan co-expression network. **a** Distribution of rattan PCC values. **b** Threshold screening of PCC and MR constructed networks by ROC curve evaluation. **c** The candidate gene pairs obtained by both methods were evaluated using the ROC curve. (The top is *C. simplicifolius* and the bottom is *D. jenkinsiana*) **d** The summary of co-expression networks in rattan

Based on the 3δ principle in developing co-expression networks with Pearson correlation coefficients (PCCs) and mutual ranks (MRs) [8], the lowest TPM values for *C. simplicifolius* and *D. jenkinsiana* were 0.16 and 0.38, respectively (Additional file 2: Fig. S3). The PCC values within genes conformed to a normal distribution (Fig. 2a), and this study extracted the top5% and bottom5% PCC gene pairs among the two rattans. Therefore, for *C. simplicifolius*, identifying the gene pairs whose PCC values fell between [0.58, 1] positively correlated with co-expression. Gene pairs whose PCC values lay within the interval [− 1, − 0.45] had a negative correlation in co-expression. Similar findings were observed in *D. jenkinsiana*, where the gene pairs with PCC values in the [0.54, 1] interval showed a positive correlation in expression. Gene pairs with PCC values in the interval [− 1, − 0.40] negatively correlated (Fig. 2a). In addition, we utilize the receiver operating characteristic (ROC) curve to evaluate the reliability of the constructed network. By screening the maximum area under the curve (AUC) based on the step size of 0.1, it was determined that the AUC value of the co-expression network was the largest at top5% + 0.2 (Fig. 2b). These values were 0.7022 and 0.6885 for *C. simplicifolius* and *D. jenkinsiana*, respectively. To further remove co-expression relationships of lower credibility, we integrated two types of MR results, i.e., unidirectional MR < 3 and bidirectional MR < 30. The results showed that the AUC values for *C. simplicifolius* and *D. jenkinsiana* were 0.8671 and 0.8601, respectively (Fig. 2b). Thus, the network constructed using the PCC and MR methods identified 10,411,977 gene pairs of *C. simplicifolius* and 10,142,311 of *D. jenkinsiana*.

Lastly, the final constructed network using the two methods above contained 49,923 genes and 39,814 genes for *C. simplicifolius* and *D. jenkinsiana*, respectively. The evaluation of the AUC value indicated that *C. simplicifolius* AUC was 0.7776, and *D. jenkinsiana* AUC was 0.7408 (Fig. 2c). The two methods covered 97.66 and 79.34% of the genes, respectively, with gene coverage enhanced by 35.50 and 10.41%, respectively [8]. The AUC values were improved by 8.60 and 7.34%, respectively [8]. The average gene connectivity of the co-expression networks of *C. simplicifolius* and *D. jenkinsiana* increased by 1159.1 and 423.9, respectively (Fig. 2d).

### Evaluation of the rattan co-expression network

**Fig. 3:**
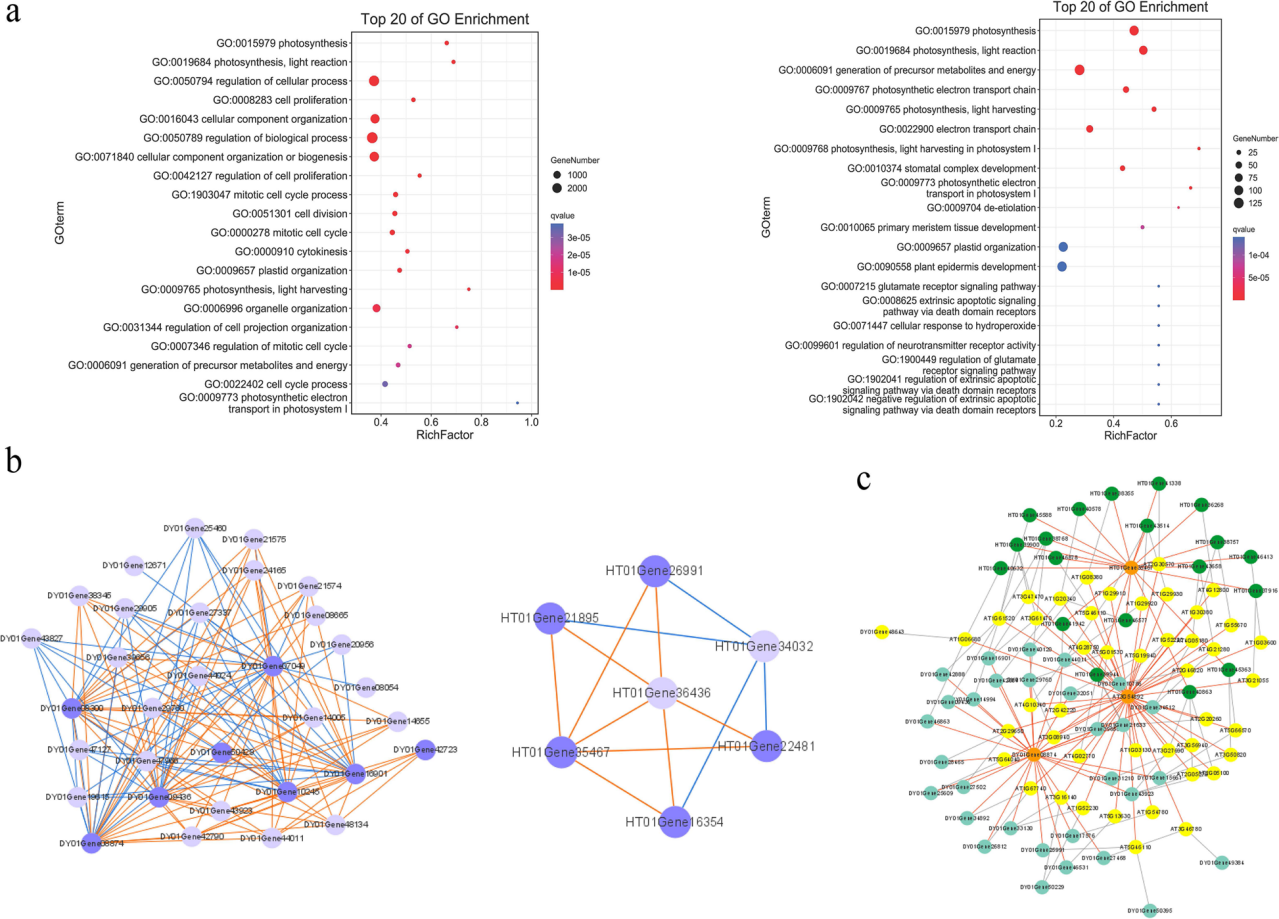
Assessing co-expression network reliability using the *LHC* gene family. **a** GO enrichment of co-expressed genes in *LHC* of rattan. (The left is *C. simplicifolius,* and the right is *D. jenkinsiana*) **b** Co-expression link between the *LHCA*s and *LHCB*s of rattan. The dark nodes indicate *LHCA* genes, light nodes indicate rattan’s *LHCB* genes, orange lines indicate positive regulatory relationships between co-expressed gene pairs, and blue lines indicate negative regulatory relationships between co-expressed gene pairs. **c** Comparison of PCC top 300 co-expression genes in rattan and *Arabidopsis LHCA1*. The dark green node indicates *D. jenkinsiana*. The light green node indicates *C. simplicifolius*. The yellow node indicates *Arabidopsis thaliana*. The orange node indicates the *LHCA1* gene of each species, the red line indicates the co-expression relationship, and the grey line shows the direct homologous relationship

We evaluated the reliability of the constructed co-expression network from three different perspectives. Since photosynthesis was relatively conserved across plant evolution, it was widely used to assess the reliability of co-expression networks [45]. In our study, related analysis was performed. Before evaluation, we re-identified light-harvesting complex (*LHC*) genes for photosystems I and II based on the updated annotation (Additional file 1: Table S4). The results indicated 47 and 14 homologs of the *Arabidopsis LHCs* in *C. simplicifolius* and *D. jenkinsiana*, respectively. GO terms related to photosynthesis were identified, most of which were found in chloroplasts and plastids (Fig. 3a). These results agreed with previous studies, indicating that the *LHC*’s primary function was to provide light energy for photochemical reactions [46]. The relationship between co-expressed genes implied that they may perform similar biological processes in a co-expression network [47]. The co-expressed gene enrichment analysis results were consistent with these findings, suggesting that the rattan networks were reliable.

In addition, we observed only a co-expression link between the *LHCA*s and *LHCB*s, indicating that the *LHCA*s and *LHCB*s were closely related (Fig. 3b). We found 136 co-expressed edges between 24 *LHCB*s and 8 *LHCA*s, with an average of 4.25 co-expressed gene pairs per gene in *C. simplicifolius*. *D. jenkinsiana* also exhibited 14 edges representing co-expression of 2 *LHCA*s and 5 *LHCB*s, with an average of two co-expressed gene pairs per gene. All *LHCA*s and *LHCB*s were co-expressed in the two rattans, suggesting that they may play a significant part in photosynthesis. In contrast to moso bamboo with on average 1.3 co-expressed gene pairs per gene [19], the expression of *LHCA*s and *LHCB*s in the two rattans was more closely connected. It indicated that the co-expression network established by the rattan could reveal the connection between *LHCA*s and *LHCB*s to the greatest extent; that is, the networks we constructed were relatively reliable.

Furthermore, we compared the PCC top300 co-expression networks of the *LHCA1*s of the two rattans with the PCC top300 ones of the *Arabidopsis LHCA1*s. These results indicated a high degree of concordance and homolog pairs were found in the two networks. As shown in Fig. 3c, there were 45 and 31 pairs of direct homologs with the *Arabidopsis LHCA1* gene in *C. simplicifolius* and *D. jenkinsiana*, respectively. This suggested that the co-expressed genes of *LHCA1* were conserved, and the light-harvesting complex genes were determined from the co-expressed genes of *LHCA1* in the three species. This was in line with the finding that *LHCA*s exhibit an aggregating pattern [32]. Thus, the above three aspects of network reliability analysis indicated that the co-expression network constructed in the two rattans was highly reliable and can be used further to analyze other related genes.

### Analysis of the NAC and MYB gene families in the rattan

**Fig. 4:**
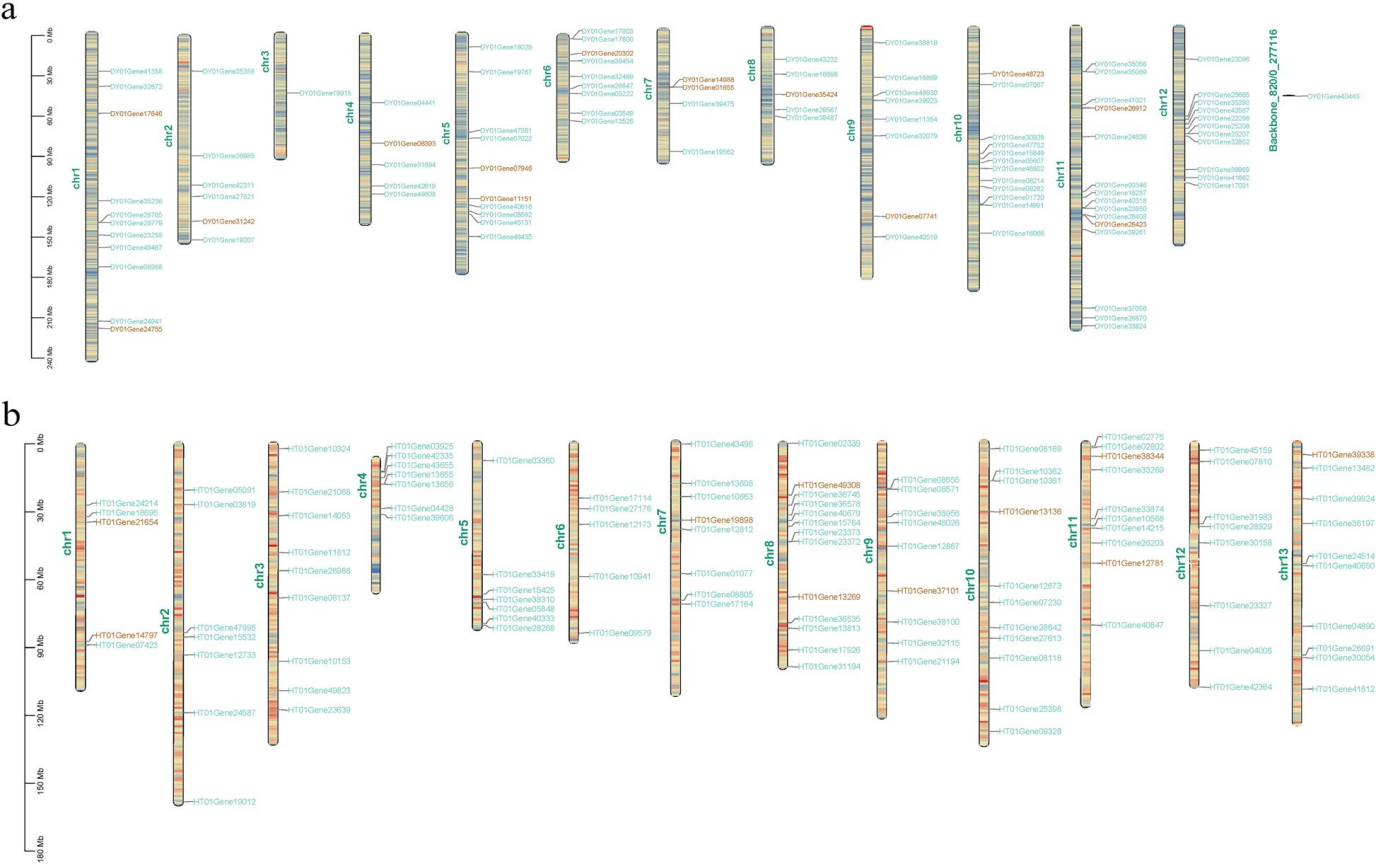
Chromosomal mapping of rattan *NAC*s and *MYB*s. **a** *C. simplicifolius*. **b** *D. jenkinsiana*

Based on the genome-wide re-annotation, we re-identified the *NAC* and *MYB*, containing 14 and 12 *NAC*s and 85 and 107 *MYB*s in *C. simplicifolius* and *D. jenkinsiana*, respectively (Additional file 1: Table S5 and S6). The results of the mapping to chromosomes indicated that the distribution of the two gene families on the chromosome was uneven (Fig. 4). The gene density on Chr11 in *C. simplicifolius* (Fig. 4a) was the highest, with two *NAC*s and 13 *MYB*s. Chr13 had the smallest number of genes, containing only one *MYB*. The results also indicated two *NAC*s located on Chr7 of *C*. *simplicifolius,* resulting from tandem repeats. Because of tandem duplication, there are 12 *MYB*s in *C. simplicifolius* and 16 *MYB*s in *D. jenkinsiana* (Fig. 4b). Two repeated *MYB*s were detected on chromosomes 1, 6, 10, and 12, respectively, and four tandemly repeated *MYB*s on Chr11 of *C. simplicifolius*. In *D. jenkinsiana*, 4 *MYB*s tandem repeat genes were found on Chr4, and 2 *MYB*s tandem repeats were found on chromosomes 5, 8, 9, and 11, respectively.

We also investigated the intron and exon structure patterns by aligning their gene sequences and CDS to understand better the similarities and differences between the structures of the two gene families (Additional file 2: Fig. S4). For the *NAC* gene family of *C*. *simplicifolius*, there are 12 genes with 3 CDS regions, one with 6 CDS regions, and one with 5 CDS regions (Additional file 2: Fig. S4a). There are five genes with four CDS regions, one gene with six CDS regions, and six with three CDS regions in *D. jenkinsiana* (Additional file 2: Fig. S4b). The genes of the same subfamily generally had a similar number of intronic areas in both rattans. Additionally, conserved domains illustrated that 26% of the MYBs in *C*. *simplicifolius* had the PLN03212 (MYB5) domain, 4 MYBs had an REB domain, 1 MYB had a Myb_6 domain, and 2 MYBs had a Myb_Cef domain. 36.37% of the final MYBs have binding sites for MYB transcription factors, and 63.53% of MYBs had PLN03091 domains. *DY01Gene38969* had a part that interacts with ATPase, suggesting that it was involved in the power supply process. Moreover, all 14 NACs in *C. simplicifolius* contained NAM domains. 28.97% of the MYBs in *D. jenkinsiana* had the PLN03212 (MYB5) domain. An estimated 37.38% of MYBs had binding sites with MYB transcription factors, and 64.45% had PLN03091 domains. Also, the *HT01Gene11812* had a domain that interacts with ATPase.

Identifying *cis*-regulatory element in promoter regions was imperative for understanding gene expression patterns. Thus, we found that the promoter regions of the two gene families had many *cis-*regulatory elements in common, including *cis-*regulatory elements related to stress, hormones, and development (Additional file 2: Fig. S4). As an abscisic acid-responsive binding motif, 51.76 and 98.1% of the MYBs had the ABRE *cis-*regulatory elements in the gene families of *C. simplicifolius* and *D. jenkinsiana*, respectively. In contrast, only 14.29% of the genes in the NACs of *C. simplicifolius* showed this pattern. This indicated abscisic acid has a more robust regulatory effect on MYBs than NACs in the two rattans. Our results indicated that 48.24% of the MYBs had low temperature-responsive *cis-*regulatory elements (LTRs) in their promoter regions, while one NAC, *DY01Gene06393*, had LTRs in its promoter regions. For *D. jenkinsiana*, 55.29% of the MYBs contained low temperature-responsive *cis-*regulatory elements (LTRs), and no LTR binding site was found in the upstream 3 kb region of the NACs. The MBS and MBSI *cis-*regulatory elements were MYB binding sites regulating drought-inducible and flavonoid biosynthesis genes, respectively. The results revealed that 30.59 and 8.24% of the MYBs had MBS *cis-*regulatory elements, and 2.45 and 12.94% of the MYBs had MBSI *cis-*regulatory elements in *C. simplicifolius* and *D. jenkinsiana*, respectively. Furthermore, most of the *cis-*regulatory elements of NACs and MYBs in the rattan were associated with development and light response. There are also other cis-regulatory elements, such as the endosperm, root, seed, and circadian rhythm.

### Expression profiling of the NAC *and* MYB gene families

**Fig. 5:**
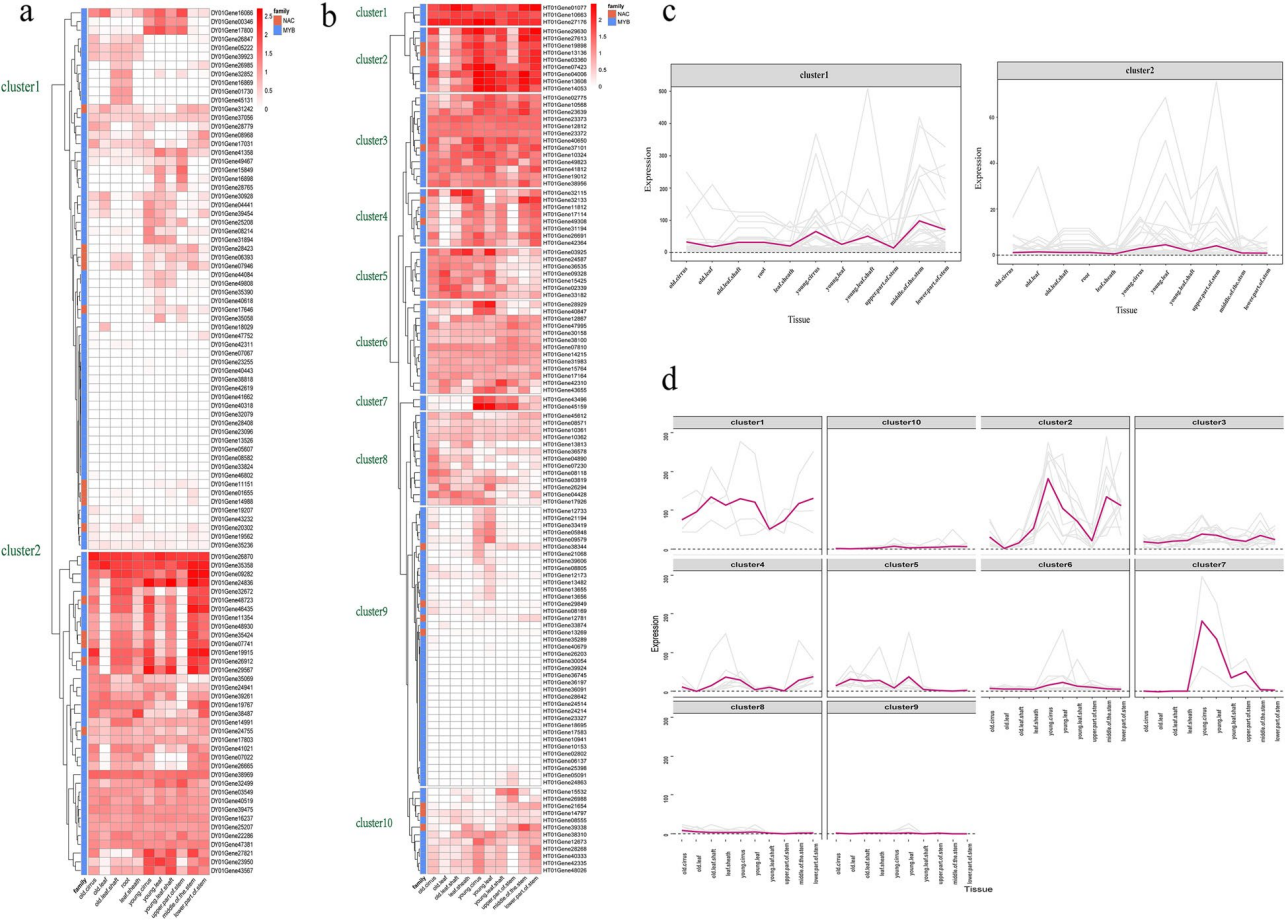
Heatmap and cluster analysis of the expression of *NAC*s and *MYB*s in rattan. **a** and **b** The heatmap of gene expression in *C. simplicifolius*. and *D. jenkinsiana*. c and d. Gene expression pattern clustering map in *C. simplicifolius* and *D. jenkinsiana*

Based on the above characterization of DNA sequences, we further analyzed the expression of *NAC*s and *MYB*s in different tissue samples in the rattan (Fig. 5). The gene expression heatmap of *C. simplicifolius* exhibited genes in both gene families exhibited similar expression patterns, and 5 *NAC*s and 37 *MYB*s were highly expressed in the different tissues (Fig. 5a). The *MYB*s were expressed in the upper portion of stem and leaf with low abundance. By combining gene expression cluster analysis with the gene expression heatmap, the genes of *C. simplicifolius* were classified into two clusters. The genes in Cluster1 (Fig. 5c) were expressed at low level in old leaf, leaf sheaths, young leaf, and the upper part of stem. A high expression level was observed in the young leaf shaft, young cirrus, and the middle of the stem. Cluster2 was the reverse; the expression level was high at the upper of stem and young leaf, whereas the expression level was low in the middle of stem and young leaf shaft. Additionally, genes in both clusters were not as highly expressed in old and root tissues as they were in young ones.

We also observed tandem repeats in gene expression patterns of *NAC*s and *MYB*s. The results demonstrated that two tandem repeat genes on the same chromosome were members of Cluster1 and shared similar gene expression patterns. There are, for example, both *MYB*s (*DY01Gene14988*, *DY01Gene01655* and *DY01Gene28408*) and *NAC* (*DY01Gene28423*) that were located on chromosomes 7 and 11 (Fig. 4), respectively. However, two tandem repeat *MYB*s on the same chromosome belong to different clusters and have opposite expression patterns, i.e., *DY01Gene25208* and *DY01Gene25207*, *DY01Gene17800* and *DY01Gene17803*, *DY01Gene01730* and *DY01Gene14991*, *DY01Gene35058* and *DY01Gene35069* on chromosomes 2, 6, 10, and 11, respectively (Fig. 4).

A similar analysis was carried out on *D. jenkinsiana*, and the results showed that five *NAC*s were highly expressed in various tissues (Fig. 5b). According to their differential expression levels, genes were grouped into 10 clusters (Fig. 5d). The expression level of the genes in Cluster1 was as high as about 100 (calculated by TPM) in the old leaf shaft, young cirrus, young leaf, leaf sheaths, and stem but was lower in the young leaf shaft. The gene expression level in Cluster1 was higher than in other clusters in different tissues. The gene expression levels of Cluster8, Cluster9, and Cluster10 were nearly zero in various tissues. In young cirrus, TPM of Cluster2 and Cluster7 tended to approach 200. Cluster2 was highly expressed in the middle of the stem, while cluster7 was more prominent in the upper portion.

Similarly, we observed that the *MYB*s tandem repeat *HT01Gene03925* and *HT01Gene42335* on Chr4 belong to Cluster5 and Cluster10. *HT01Gene03925* was highly expressed in young leaf and young cirrus. Two *MYB* tandem repeat genes (*HT01Gene08555* and *HT01Gene08571*) on Chr9 were grouped in Cluster8 and Cluster 10, respectively, and there was nearly no gene expression from these clusters. Other *MYB*s tandem repeat genes located on different chromosomes were organized into one cluster. For example, *HT01Gene13655* and *HT01Gene13656* of Cluster9 were located on Chr4. *HT01Gene4033*3 and *HT01Gene2868* of Cluster10 were located on Chr5. *HT01Gene23373* and *HT01Gene23372* of Cluster3 were located on Chr8. *HT01Gene10362* and *HT01Gene10361* of Cluster8 were located on Chr10. Hence, *NAC*s and *MYB*s were higher in young rattan tissues than in old tissues, and the expression of *NAC*s and *MYB*s was more active in young leaf. It was consistent with the habit of climbing rattans to capture light sources as they grew up [3]. Furthermore, the genes were differentially expressed in different parts of the stem, suggesting that NAC and MYB transcription factors may be involved in rapid growth in the rattan stem. Rattan tandem repeat *NAC* and *MYB* displayed similar expression patterns and opposite expression trends.

### Co-expression analyses of the NAC, MYB, and lignin biosynthetic pathways

**Fig. 6:**
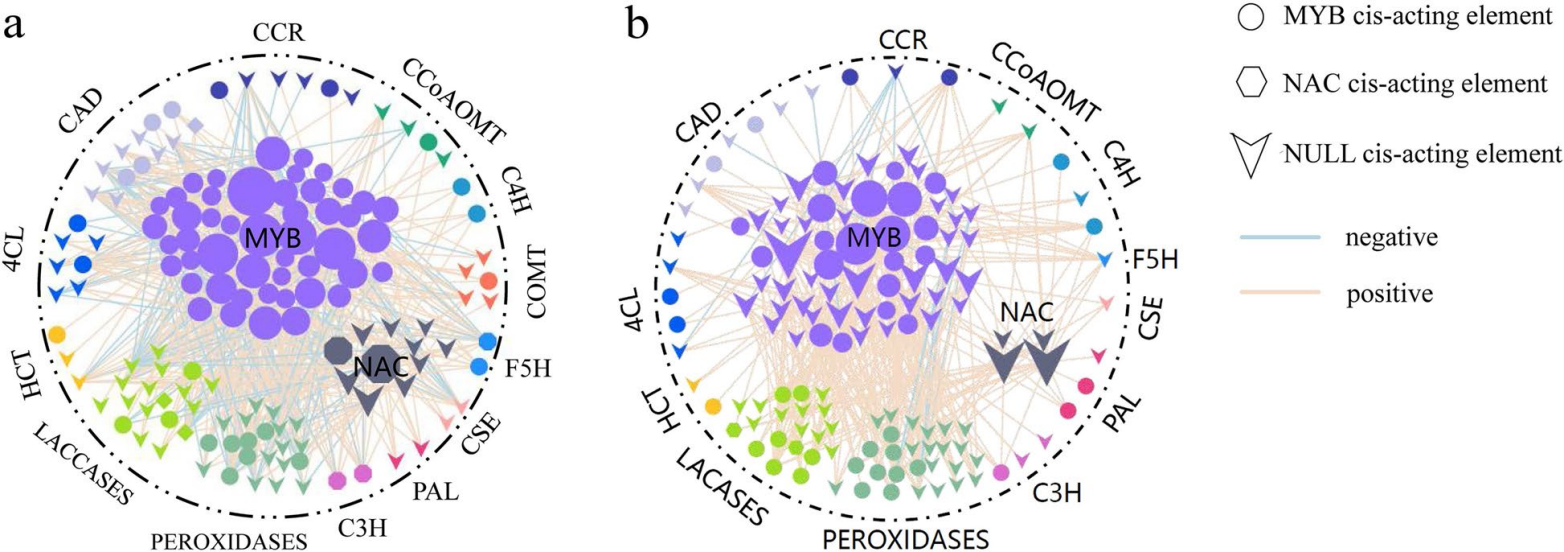
Co-expression genes of NAC and MYB were involved in the lignin biosynthesis pathway. **a** *C. simplicifolius*. **b** *D. jenkinsiana*

An analysis of the lignin biosynthesis pathway (Additional file 1: Table S7) of the co-expression genes of NAC and MYB in both rattans was performed. For *C. simplicifolius*, 11 NACs and 49 MYBs co-expressed in the lignin biosynthesis pathway were used to construct a co-expression network (Fig. 6a), including 146 network nodes, 520 co-expression edges, and average gene connectivity of 7.123. There were 91 co-expression pairs between 11 NACs and 40 lignin biosynthesis genes, of which 20.88% were negatively correlated, and the rest were positively correlated. Additionally, 12 gene families involved in the lignin biosynthesis pathway were co-expressed with these 11 NACs, suggesting that NACs were profoundly involved in regulating the lignin biosynthesis pathway. Among these 40 genes, the *LACCASES* gene family (which accounted for 20%) and the *PEROXIDASES* gene family (27.5%) accounted for the largest proportions. Among these 91 co-expression pairs, several genes appeared to have a close relationship to the development of wood properties. For example, *DY01Gene07741* (SND2*/*SND3) had the highest degree of connectivity, and there was an interactive relationship with 22 genes. Interestingly, this gene had a homolog of *Arabidopsis* NAC073, which regulated the expression of genes involved in cellulose and hemicellulose biosynthesis, lignin polymerization, and signaling [48]. Another example was that the *DY01Gene11151* (VND1*/*VND2) had 16 co-expression edges in the network. The gene had a homolog of *Arabidopsis* NAC037, which was involved in the xylem biosynthesis pathway [31].

We identified 430 pairs of co-expression relationships between 49 MYBs and 81 genes in the lignin biosynthesis pathway of *C. simplicifolius* (Fig. 6a), of which 33.95% were negative, and the rest were positive correlations. The lignin biosynthesis pathway was co-expressed with 49 MYBs, indicating that MYBs are active in regulating the lignin biosynthesis pathway. Among the 81 genes that interact with the lignin biosynthesis pathway, members of the *LACCASES* gene family (21%) and the *PEROXIDASES* gene family (23.46%) accounted for the largest proportion. Among the 430 co-expression pairs, *DY01Gene08968* had the highest degree of connectivity, and there were interactions with 22 genes. The ones with higher connectivity include *DY01Gene28765* (connectivity 18), *DY01Gene17031* (connectivity 18), and *DY01Gene15849* (connectivity 17). 4.76 and 27.38% of the genes in the lignin biosynthesis pathway in the constructed co-expression network contained binding sites for NAC transcription factors and MYB transcription factors, respectively. Our research revealed that three genes (*DY01Gene26809*, *DY01Gene19156*, and *DY01Gene21671*, belonging to the *CAD* and *LACCASES* gene families) had both NAC and MYB binding sites, suggesting that both NAC and MYB regulated these genes.

A similar analysis was carried out in *D. jenkinsiana*, and the results showed co-expression genes of the lignin biosynthesis pathway, including four NACs and 59 MYBs. We constructed a co-expression network of the lignin biosynthesis pathway in *D. jenkinsiana*, containing 140 network nodes, 297 co-expression edges, and average gene connectivity of 4.243 (Fig. 6b). The network results indicated that four NACs formed 55 pairs of positive co-expression relationships with 21 genes in the lignin biosynthesis pathway. The four NACs were co-expressed with ten gene families in the lignin biosynthesis pathway: *C3H*, *CAD*, *CCoAOMT, CSE*, *F5H, C4H*, *CCR*, *PAL*, *LACCASES,* and *PEROXIDASES*. The co-expression relationship of NAC indicated that *HT01Gene13136* (SND2*,* SND3) had a co-expression relationship with 19 genes, making it the gene with the highest degree of connection in the co-expression relationship, and it was a homolog of *Arabidopsis* NAC073. Additionally, *HT01Gene19898* (NST1, NST2, SND1, NST3) contained 16 mutual edges, a homolog of NAC043 in *A. thaliana*. NAC073 and NAC043 were involved in lignifying secondary cell walls [30].

Furthermore, we found 337 pairs of co-expression relationships between 59 MYBs and 76 genes in the lignin biosynthesis pathway of *D. jenkinsiana* (Fig. 6b), of which 5.64% were negatively remainder were positively correlated. Among the co-expression genes of the lignin biosynthesis pathway, 26.32% were members of the *LACCASES* gene family, and 32.89% were members of the *PEROXIDASES* gene family. For example, *HT01Gene17114* (MYB103) was the gene with the highest degree of connectivity in the co-expression network of MYBs, with 20 interactions, followed by *HT01Gene03360* (MYB42) (connectivity 15), *HT01Gene04006* and *HT01Gene08169* (connectivity 12), and *HT01Gene27613* (connectivity 11). In addition, the genes involved in the lignin biosynthesis pathway co-expressed with both MYB and NAC belong to 12 gene families. A total of 2.74% of these genes had binding sites for NAC transcription factors that belong to the *LACCASES* gene family. Additionally, 35.62% of these genes had binding sites for MYB transcription factors, including the *4CL*, *C3H, C4H*, *CAD*, *CCR*, *LACCASES*, *HCT,* and *PEROXIDASES* gene families.

**Fig. 7:**
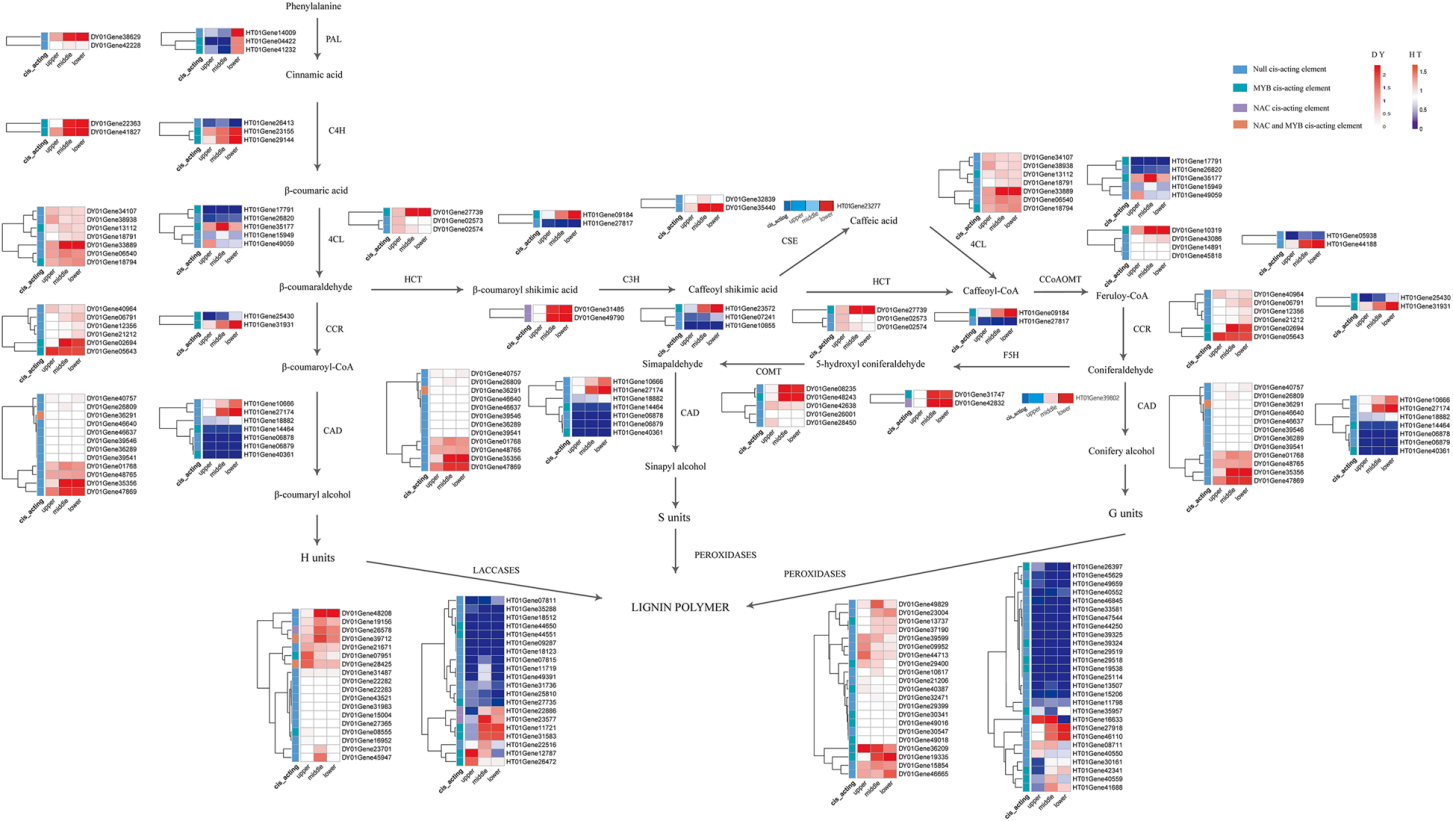
An overview of the co-expression of NACs and MYBs in the lignin biosynthesis pathway

We investigated the expression of genes co-expressed by NAC and MYB in the lignin biosynthesis pathway of rattan stem tissue (Fig. 7). Several gene families were found in the middle or base of the stem in both rattans, which indicated lignin deposition during plant growth [49]. Combined with the lignin biosynthesis pathway network of the co-expression genes (Fig. 6), we found 157 pairs of lignin biosynthesis genes with high expression in the rattan. In particular, 15.29% of the gene pairs were co-expressed with MYB83, MYB46, MYB32, MYB7, MYB4, MYB43, and 25.48% of the gene pairs were found to be co-expressed with SNDs, VNDs, and NSTs. Furthermore, 38 lignin biosynthesis genes were not detected to be expressed, and 172 pairs of co-expression were identified. 16.18% of lignin biosynthesis genes were co-expressed with SNDs, VNDs, and NSTs, and 6.74% were co-expressed with MYB83, MYB46, MYB32, MYB7, MYB4 and MYB43. The abovementioned results implied that the highly expressed genes and the non-expressed lignin biosynthesis genes in the stem tissue of *C. simplicifolius* were positively and negatively regulated by NAC, respectively.

Additionally, as a result of co-expressed genes of NACs and MYBs in *D. jenkinsiana*, 85 pairs of genes with high expression were identified in the lignin biosynthesis pathway, of which 28.24% were co-expressed with SND, VND, and NST genes, and 25.88% were co-expressed with MYB42 and MYB103. There were 104 co-expression pairs among the 29 lignin biosynthesis genes, and no genes were found to co-express with SND, VND, or NST. Furthermore, we also found that 4.80% of the gene pairs co-expressed with MYB58, MYB20, MYB43, MYB32, MYB63, MYB4, and MYB7, suggesting that MYB in *D. jenkinsiana* involved in the process of non-expressed lignin biosynthesis genes in stem tissue.

**Fig. 8:**
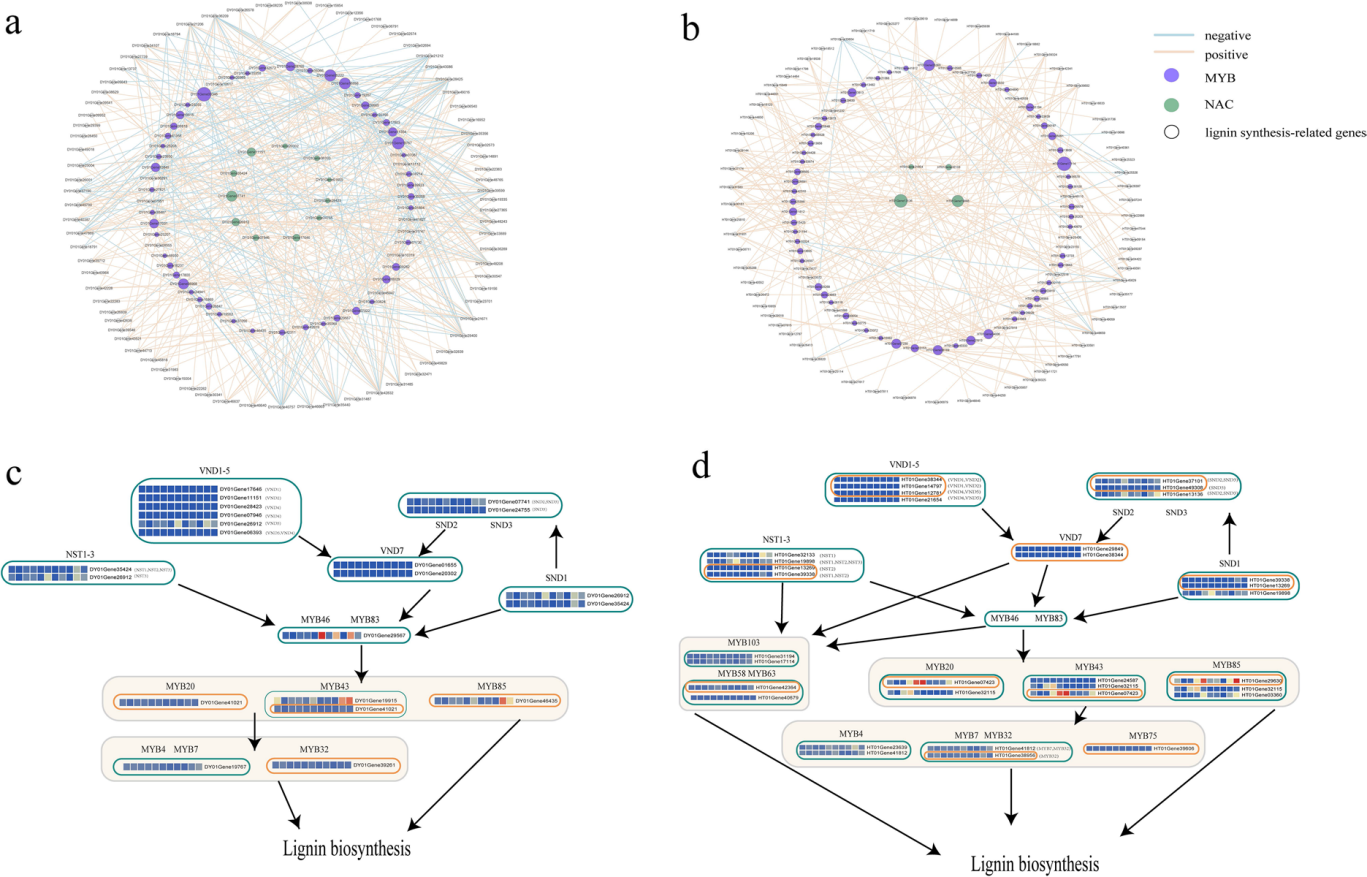
Rattan co-expression and regulatory network for NAC and MYB*.* **a** and **b** Regulatory network of NAC, MYB and lignin synthesis-related genes in *C. simplicifolius* and *D. jenkinsiana*. c.and d. NAC and MYB core regulatory network in *C. simplicifolius* and *D. jenkinsiana*. Green and orange boxes indicate the co-expressed genes of NAC and MYB involved in lignin biosynthesis versus those not involved

Finally, according to the network for the lignin biosynthesis pathway based on NAC and MYB (Fig. 8a and b)*,* we constructed a core regulatory network of NAC and MYB in both rattans (Fig. 8c and d). We found that the results of the core regulatory network of *C. simplicifolius*, MYB43, MYB20, and MYB85 exhibited similar expression patterns (Additional file 2: Fig. S5), *DY01Gene01655 (*VND7*)* regulated only one lignin biosynthesis gene, *DY01Gene32471* and *DY01Gene19767 (*MYB4*/*MYB7*)* regulated 78% of the lignin biosynthesis genes (Fig. 8a). As a result of the core regulatory network analysis in *D. jenkinsiana*, *HT01Gene32115* was identified as MYB20, MYB43*,* and MYB85 by homolog analysis. Nevertheless, *HT01Gene32115* and other MYB20, MYB43*,* and MYB85 were differentially expressed in young cirrus and old leaf shaft tissues (Fig. 8d), suggesting that they may perform different biological functions. Furthermore, *HT01Gene17114* (MYB103) and *HT01Gene13136* (SND2*/*SND3) regulated 27.63 and 25% of lignin biosynthesis genes (Fig. 8b), respectively, suggesting both genes may act as hub genes in the network.

## Discussion

In 2018, our team published the genome sequences and gene annotations for both rattans (i.e., *C. simplicifolius* and *D. jenkinsiana*), which provided critical primary data for the molecular research of rattan and other plants. With the development of omics technologies, there are more demands on bioinformatics data, both in terms of quality and quantity. Until recently, the genetic information of the rattan has been continuously improved, contributing to the advancement of research in this area. This study utilized a large amount of transcriptome data and an optimized annotation process to genome-widely re-annotate the genes in both rattans. The process not only corrects some errors but also detected more new gene models. As a result, this research will advance in-depth research on functional genes in rattan. Additionally, we employed a newly developed pipeline of function annotation. As can be seen from the results, the updated version of the annotation has improved by 3–4% based on the high proportion of the previous annotation (93–94%). This may be because the updated version used more databases and the data for each database was continuously increasing.

We improved a co-expression network construction pipeline, which has significantly enhanced gene coverage and the reliability of the co-expression network. Currently, co-expression network was constructed by two methods, i.e., PCC & MR [8] and WGCNA [50], but each had certain limitations. PCC and MR approach considered the degree of linear correlation, and the resulting threshold was hard thresholding [50]. WGCNA considered direct and indirect correlations between genes, and the resulting threshold was soft thresholding [51]. Thus, we integrated the screening strategies of both methods into the threshold and developed a new pipeline for constructing co-expression networks by obtaining a “half-threshold” (i.e., can absorb the advantages and disadvantages of soft and hard thresholds) from the perspective of robustness when building the network. It helps to retrieve some gene co-expression lost in the hard point. The gene pairs obtained through soft thresholding were filtered, although the strongly correlated gene pairs were retained. Thus, in the new approach, we can more accurately represent the interaction information between genes for studies with low to medium sample sizes (e.g., greater than 15 samples of transcriptome data, this is mainly due to the WGCNA sample requirements) and better to understand the co-expression relationships between genes in organisms.

To assess the reliability of co-expression networks, we used light-harvesting gene families. We selected the top three matching proteins as the optimal homologous genes based on a homologous alignment of all protein sequences from both rattans with that of the *Arabidopsis LHC* gene. The analysis method has been widely used in many studies [8, 10, 19]. Thus, 47 and 14 *LHC*s were identified for *C. simplicifolius* and *D. jenkinsiana*, respectively (Additional file 1: Table S4). Also, we observed the differences in the number of *LHC* identified between both rattans. According to our preliminary observations and hypothesis, it may be due to the two rattans’ different growth habits and the positions of the cirrus. It is well known that rattans use cirrus to help them climb to gain more light. There are two types of cirrus. One was the cirrus with spines extending from the leaf axis, called the “leaf cirrus.” The other was the cirrus on the leaf sheath, designated the “sheath cirrus” [52]. There are also differences in function and structure, and because of this difference in structure, both rattans differ slightly in their climbing habits [52]. Therefore, the difference in homologous genes results may be species-specific, which is well worth further investigation.

In evaluating co-expression network reliability using photosynthesis, we investigated the expression of the light-harvesting complex gene (*LHC*) in different tissues of the rattan (Additional file 2: Fig. S6). The results indicated that these genes were expressed in old leaf, leaf sheaths, and cirrus. This was consistent with the habit of rattans, the typical companion trees, of climbing stems to capture more light [52]. The expression of these genes was not observed in roots compared to other tissues. Similar to *D. jenkinsiana*, it was determined that the *LHC*s were expressed in the old leaf shaft and young leaf. The difference in expression of the *LHC*s between the two rattans might be attributed to their different climbing habits and the position of their cirrus. There were two kinds of cirrus on rattans. One was the cirrus with spines extending from the leaf axis, called the “leaf cirrus.” The other was the cirrus on the leaf sheath, designated the “sheath cirrus” [52]. Moreover, the two rattans differed in function and structure, and the difference in the structure caused the rattans to exhibit quite different climbing habits.

In the analysis of NAC and MYB in rattan, we found that NACs and MYBs control the genes involved in the lignin biosynthesis pathway for both rattans to varying degrees. The findings were consistent with previous research [21, 53]. However, we found that the *LACCASES* and *PEROXIDASES* gene families displayed the most extensive co-expression relationship with NAC and MYB (20%), which was likely because these genes were in the last steps of the lignin biosynthesis pathway. In *C. simplicifolius*, the gene families expressed by NAC contained *C3H*, *CAD*, *F5H*, and *LACCASES*. In contrast, the gene families regulated by MYB included *4CL*, *C4H, CAD*, C*CoAOM*T, *CCR*, *COMT*, *F5H*, *HCT*, *LACCASES*, and *PEROXIDASES*; of these *CAD* and *LACCASES* were regulated by both MYB and NAC. In *D. jenkinsiana*, the gene family regulated by NAC was *LACCASES*. The gene family regulated by MYB was *4CL*, *C3H*, *C4H*, *CAD*, *CCR*, *LACCASES*, *HCT*, *PAL,* and *PEROXIDASES*; both NAC and MYB regulated *LACCASES*.

## Conclusions

In this study, we re-annotated the genomes and enhanced the gene annotation accuracy for both rattans. Based on developing a pipeline for building co-expression networks, we improved gene coverage and the reliability of the co-expression network. We focused on the regulatory mechanisms of NAC and MYB transcription factors in the rattan’s lignin biosynthesis pathway. Thus, this study will provide new insight into gene annotation and co-expression networks and identify and reveal key NAC and MYB transcription factors in rattan and their regulatory mechanisms.

## Methods

### Sample description

To construct a comprehensive network, 35 and 46 samples from young and old stages were collected from *C. simplicifolius* and *D. jenkinsiana* at the Research Institute of Tropical Forestry of the Chinese Academy of Forestry in the city of Guangzhou (Additional file 1: Table S8). The samples were taken at different developmental stages and from other tissues. Prof. Rongsheng Li identified these field samples, and sample resources were deposited in the Institute of Tropical Forestry. (To ensure reproducibility, we provide data resources for all samples, as described in the availability of data and materials section of the statement.). In addition, the sample collection process was permitted and supported by the Institute of Tropical Forestry and did not destroy other species’ resources, in compliance with local laws and regulations. And we comply with the IUCN Policy Statement on Research Involving Species at Risk of Extinction and the Convention on the Trade in Endangered Species of Wild Fauna and Flora. To isolate RNA, we used TRIzol Reagent (Invitrogen, Carlsbad, CA, United States) and a NanoDrop 2000 spectrophotometer (instructed by the manufacturer and Thermo Fisher Scientific, Waltham, MA, United States) to determine the purity and concentration of the RNA. A reverse transcription system was used to reverse transcribe the RNA (Promega, Madison, WI, United States). As described previously [47], RNA was treated with RNase-free DNase I for 30 minutes at 37 °C to remove residual DNA before reverse transcription. A BGISEQ-500 platform (Beijing Genomics Institute, Shenzhen, China) was used to sequence the pooled libraries. Moreover, data sets from the same tissue were merged for further analysis.

### Data processing and gene expression profile analysis

The transcriptomic datasets were preprocessed using FastQC v0.11.6 [54] and the default parameters for quality statistical analysis. Trimmomatic v0.36 [37] filtered the adapters and low-quality sequences using the following parameters: LEADING:3, TRAILING:3, SLIDINGWINDOWS 4:10, MINLEN:36, and TOPPHRED33. For data mapping, Trimmomatic data has been aligned with the relevant reference genome [7] using HISAT2 v2.1.0 [55] as described below with the following modifications compared to default parameters: –min-intronlen 20, −max-intronlen 4000, and –rna-strandness RF. All transcript assembly datasets from HISAT2 have been reserved (Additional file 1: Table S2). To calculate the transcripts per kilobase of exon model per million mapped reads (TPM) values, we used StringTie v1.3.5 [56] with the default parameters, except for a single additional parameter, −u. Additionally, the 3σ criterion formula of “threshold = average (5% value) + 3·D” [10] was used to calculate the TPM thresholds in each experimental group. Each gene with a TPM value below the threshold in each sample was removed due to an insignificant correlation coefficient.

### Genome annotation

For repeat annotation, tandem Repeats Finder v4.09 [57] was used to scan the genome for tandem repeats with a period size> 50 bp. A combination of de novo and homology-based approaches were used to identify transposable elements (TEs). The de novo repeat library was constructed using RepeatModeler v2.0.1 [58] with LTRStruct as a parameter. To map our assembly against the TE sequence in the repeat library and classify TEs, we used the Repbase v21.12 [59] database and RepeatMasker v4.1.0 [60]. Wu-BLASTX [61] was applied to the TE protein database in RepeatProteinMask v4.0.7 [60] to identify TEs at the protein level.

We performed integrated predictions on the complete protein-coding gene model using three independent approaches: homologous sequence-based methods, RNA-Seq method, and ab initio predictions. The homologies from 7 species (maize (Ensembl-18), rice v7, *Brachypodium* v3.1, foxtail millet v2.2, *Arabidopsis* (TAIR10), sorghum v3, oil palm (EG5)) were used as protein evidence for predicted gene sets using GeneWise v2.4.1 [62]. A transcriptome is a data derived from the above data processing, including multiply RNA-Seq. Ab initio gene prediction was performed with AUGUSTUS v3.3.1 [63], trained by the transcriptome data. Afterward, a pipeline (https://github.com/chenlianfu/geta) was used to integrate the gene annotation results from homology-based, transcriptome-based, and ab initio predictions to generate a comprehensive protein-coding gene set. Finally, a non-redundant, consensus protein-coding gene set was constructed. Additionally, functional gene annotation was performed by searching against the authoritative databases, including Nr (release 20,210,824), Swiss-Port (release 20,210,521), COG (releases 20,210,501), EggNOG (release 20,190,630), InterPro v5, Pfam v34.0, GO (releases 20,210,501) and KASS v2.1. Thus 97.22 and 97.64% of the genes were functionally annotated in *C. simplicifolius* and *D. jenkinsiana* (Additional file 1: Table S9).

### Co-expression networks construction

Two strategies were integrated to construct co-expression networks (CENs), one developed by our team based on Pearson correlation coefficients (PCCs) and mutual ranks (MRs) [64] and the other on weighted correlation network analysis (WGCNA) [65]. The first strategy utilizes the WGCNA package in R to analyze the weighted gene co-expression network. Here are the steps: (1) Calculate the euclidean distance between samples and perform hierarchical clustering using the ward. D2 algorithm from the R language hclust function. All parameters are set to the default values. (2) After determining no outlier samples, use the pickSoftThreshold function (R-square = 0.90) in the WGCNA package to judge and select an appropriate β value that conforms to the scale-free network distribution. That is to say, the correlation between every two genes is taken to the power of β to obtain the adjacency matrix (aij). (3) Calculate the relationship between two genes based on the adjacency matrix by summing each gene’s direct and indirect correlation. (4) Dynamic shearing algorithm, hierarchical clustering based on TOM matrix [66], construction of gene tree and division of gene co-expression network modules, parameter selection, with the following parameters: TOMtype = “unsigned”, minModuleSize = 30, reassignThreshold =0, mergeCutHeight = 0.25. Finally, the gene pairs with TOM values greater than 0.1 [8] were selected as candidate gene pairs.

Secondly, we employ a co-expression network construction pipeline based on PCCs and MRs that has been successfully applied in several studies [9, 10, 19, 58, 59]. One of the major advantages of the 3δ criterion in this method [10] is that genes whose overall expression abundance value is too low will result in very small correlation coefficients when constructing the co-expression network. Thus, using this procedure will allow the researcher to determine a threshold of TPM, thereby removing genes whose expression level is lower than that threshold in most samples, which is essential for constructing an accurate co-expression network. Following is a description of the specific construction process. (1) Calculate the PCC value between genes based on the TPM value of each gene. (2) Eliminate the weakly correlated gene pairs and retain only the highly correlated gene pairs. The top 5% and bottom 5% PCC values in this study were used. These gene pairs should be retained as strongly correlated. (3) Calculate the MR value of each gene pair based on the PCC value, then isolate high-confidence gene pairs and eliminate unreliable gene pairs. MR refers to gene A to gene B and the geometric mean of PCC grades from gene B to gene A. (4) We retained gene pairs with MR values less than 30 and a unidirectional ranking of less than 3 to construct the entire co-expression network.

Integrate the above two methods to construct co-expression networks (Additional file 2: Fig. S7). To evaluate the reliability of the correlation between genes in a co-expression network, each part uses a receiver operating characteristic curve (ROC) [67]. The larger the area under the curve (AUC) is in the ROC curve analysis, the higher the confidence level of the co-expression network. Subsequently, we evaluate the network’s reliability through the above integration. The photosynthetic system light-harvesting complex gene family (*LHC*) members were identified by homologous gene identification, resulting in rattan and enriched co-expressed genes. Based on the enrichment results, the *LHC* gene family was more likely to have been involved. The more detailed the functional description, the more reliable the co-expression network will be. Additionally, we analyzed the relationship of co-expressed genes between light-harvesting complex I (*LHCA*) and light-harvesting complex II (*LHCB*). The closer the connection, the more reliable the network will be. Lastly, we compared the relationships between the two rattans and the top 300 PCC co-expressed genes of *Arabidopsis LHCA1*. Consequently, the network is likely reliable since the *LHCA* gene is conserved and the co-expressed genes of the three species are related.

PCC algorithm:

$$=\frac{\sum_{i=1}^n\left(x_i-\overline x\right)\left(y_i-\overline y\right)}{\sqrt{\sum_{i=1}^n\left(x_i-\overline x\right)^2\cdot\sum_{i=1}^n\left(y_i-\overline y\right)^2}}$$

In the PCC algorithm, *X* and *Y* are TPM values, and *n* represents the number of samples.

MR algorithm:

$$the\ MR(AB)=\sqrt{\Big( Rank\left(A\to B\right)\cdot Rank\left(B\to A\right)}$$

In the MR algorithm, *Rank (A* → *B)* is the PCC rank from gene A to gene B, and *Rank (B* → *A)* is the PCC rank from gene B to gene A.

### Genome-wide identification of NAC, MYB, and lignin biosynthesis genes in rattan

The protein sequences of *Arabidopsis thaliana* NAC and MYB TFs were downloaded from the Plant Transcription Factor Database (http://planttfdb.cbi.pku.edu.cn/index.php, accessed on 24 February 2021). Genes and coding sequences (CDSs) were also downloaded from Phytozome 12. (https://phytozome.jgi.doe.gov/pz/portal.html#, accessed on 24 February 2020). The reciprocal best hit (RBH) BLAST [61] was used to identify potential homologs proteins between the rattans and *A. thaliana* for homologs identification. In each RBH, the top three hits were selected as the best homologs pairs. Secondary homologs pairs are identified as pairs with E values less than the peak of the E-value distribution of all the best hits in *C. simplicifolius* and *A. thaliana*, *D. jenkinsiana,* and *A. thaliana*. Default parameters were used on the Pfam website to confirm the candidate NAC and MYB genes (http://pfam.xfam.org/search/keyword?query=&submit=Submit#tabview=tab1, accessed on 20 March 2020). MYB protein sequences that lack a Myb domain and NAC protein sequences without a NAM domain were manually discarded. Gene, CDS, and protein sequences of NAC and MYB families have been deposited in NCBI. The sequences of the *Arabidopsis* lignin biosynthesis genes NAC and MYB were obtained from previous studies and used as queries to identify the corresponding rattan genes. BLAST searches were performed locally with an e-value cut-off of 1e^− 100^ and 1e^− 70^ to identify NAC and MYB homologous, respectively.

### Chromosomal location and gene structure

Each *NAC* and *MYB* gene were mapped to the rattan genome using TBtools [58] with default parameters to determine its position on the chromosome. The NAC and MYB protein sequences of *rattan* were aligned using Muscle, which is built-in to MAFFT v7.475 [68]. The phylogenetic trees of NAC and MYB family members were then constructed using IQ-TREE v2.0.3 [69] and the neighbor-joining (NJ) method (bootstrap with 1000 replicates). Furthermore, all gene structures of *NAC* and *MYB* were determined by comparing the CDS sequences with their corresponding genomic sequences, to which the TBtools program was applied.

### Analysis of the promoter regions of NAC and MYB genes

The 3-kb region upstream of the transcriptional start site of NAC and MYB genes was extracted from the rattan genome to identify the cis-elements within the promoter regions. The plant Cis-Acting Regulatory Element (http://bioinformatics.psb.ugent.be/webtools/plantcare/html/, accessed on 9 May 2021) database was used to identify *cis*-elements within the promoter regions of each gene (with default parameters) and to identify the functions of each *cis*-element.

## Supplementary Information

**Additional file 1: Table S1- S9.**

**Additional file 2: Fig. S1- S7.**

## Data Availability

The datasets generated and analyzed during the current study are available in the NCBI database (National Center for Biotechnology Information; BioProject ID: PRJNA807471, PRJNA308068, PRJEB24031, and PRJEB24829). The Gene annotation file from this study has been submitted to GitHub under the accession: https://github.com/wy-cmd/Re-annotated-the-genomes-of-rattan.

## Abbreviations

4CL: 4-coumarate: CoA ligase
AUC: Area under the curve is in the ROC curve analysis
C3H: p-coumarate 3-hydroxylase
C4H: Cinnamate 4-hydroxylase
CAD: Cinnamyl alcohol dehydrogenase
CCoAOMT: Caffeoyl-CoA O-methyltransferase
CCR: Cinnamoyl-CoA reductase
CENs: Co-expression networks
COMT: Caffeic acid O-methyltransferase
CSE: Caffeoyl shikimate esterase
F5H: Ferulate 5-hydroxylase
HCT: p-hydroxycinnamoyl -CoA:quinate/shikimate p-hydroxycinnamoyltransferase
LHC: Light-harvesting complex gene family
LHCA: Light-harvesting complex I
LHCB: Light-harvesting complex II
LTR-RTs: Long terminal repeat-retrotransposons
LTRs: Low temperature-responsive cis-regulatory elements
MOE: Modulus of elasticity
MR: Mutual ranks
PAL: Phenylalanine ammonia-lyase
PCC: Pearson correlation coefficients
RBH: Best hit
ROC: Receiver operating characteristic curve
TEs: Transposable elements
TPM: Transcripts per kilobase of exon model per million mapped reads
WGCNA: Weighted correlation network analysis
