# Supplementary material for: Analyzing lignin biosynthesis pathways in rattan using improved co-expression networks of NACs and MYBs
Source: BMC Plant Biol. 2022 Aug 24;22:411. doi: 10.1186/s12870-022-03786-4 (PMC9400238; doi:10.1186/s12870-022-03786-4)
Supplement: Supplementary file 2 — Additional file 2: Fig. S1- S7. [file 12870_2022_3786_MOESM2_ESM.pdf]

Analyzing lignin biosynthesis pathways in rattan using improved co-expression networks of *NAC*s and *MYB*s

Wang *et al.*

Contents

Supplementary Figure 1. Comparison of the old and new versions of rattan annotations.

Supplementary Figure 2. Threshold, average connectivity, and module division in WGCNA results of rattan.

Supplementary Figure 3. Box plots of $\log_{10}$ (TPMs) of samples in the rattan.

Supplementary Figure 4. Gene structure and prediction of the *cis*-regulatory elements and conserved domains of the *NACs* and *MYBs* in rattan.

Supplementary Figure 5. Gene expression patterns of MYB20, MYB43, and MYB85 in *Calamus simplicifolius.*

Supplementary Figure 6. Expression profile of the *LHC* gene family in rattan.

Supplementary Figure 7. Construction and evaluation of co-expression networks.

(a)

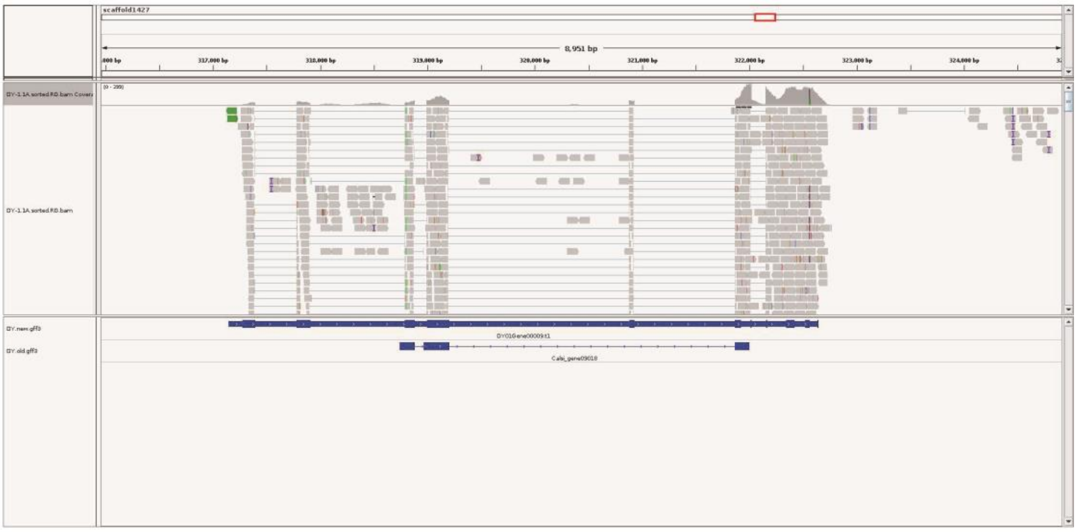

(b)

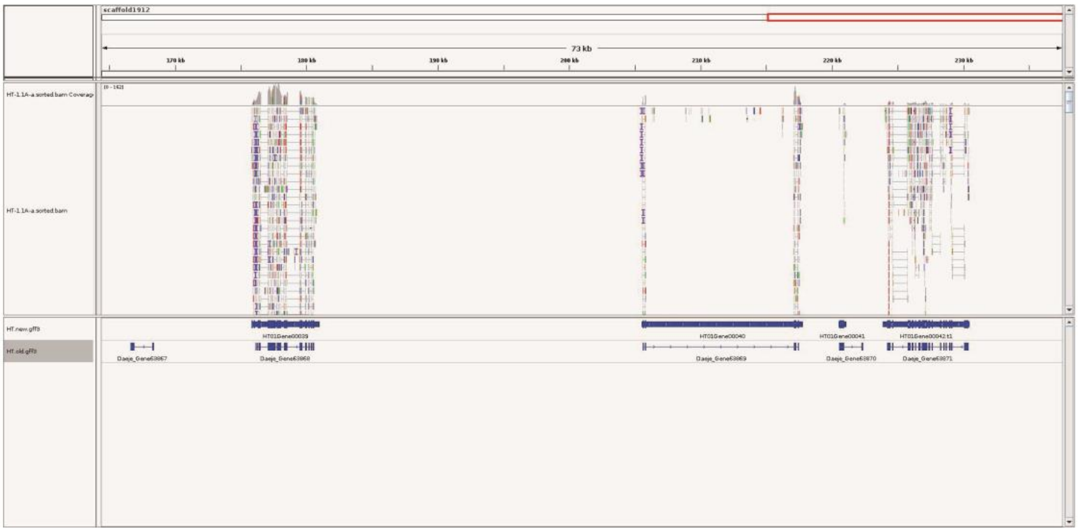

Fig. S1 Comparison of the old and new versions of rattan annotations. (a) Comparison of the old and new versions of *Calamus simplicifolius* annotations. (b) Comparison of the old and new versions of *Daemonorops jenkinsiana* annotations.

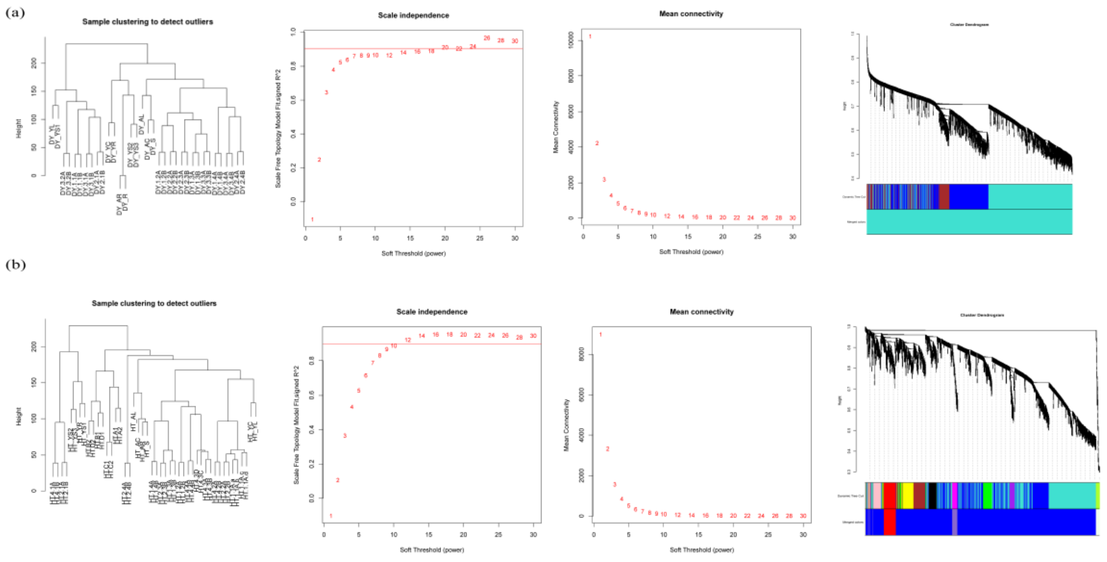

Fig. S2 Threshold, average connectivity and module division in WGCNA results of rattan. (a) The result of WGCNA in *Calamus simplicifolius*. (b) The result of WGCNA in *Daemonorops jenkinsiana*.

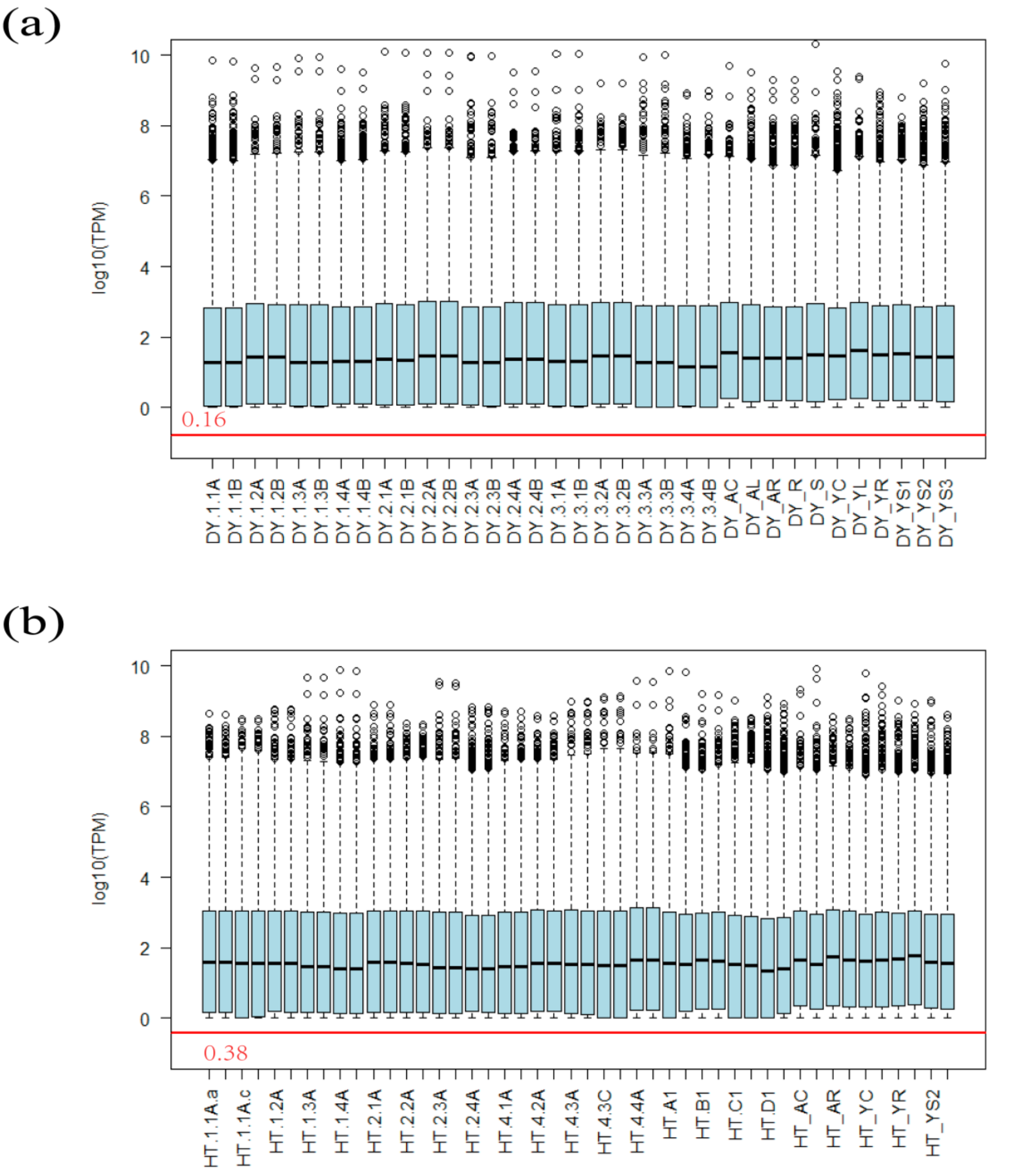

Fig. S3 Box plots of $\log_{10}$(TPMs) of samples in the rattans. (a) 0.16 is minimum TPMs of *Calamus simplicifolius*. (b) 0.38 is minimum TPMs of *Daemonorops jenkinsiana*.

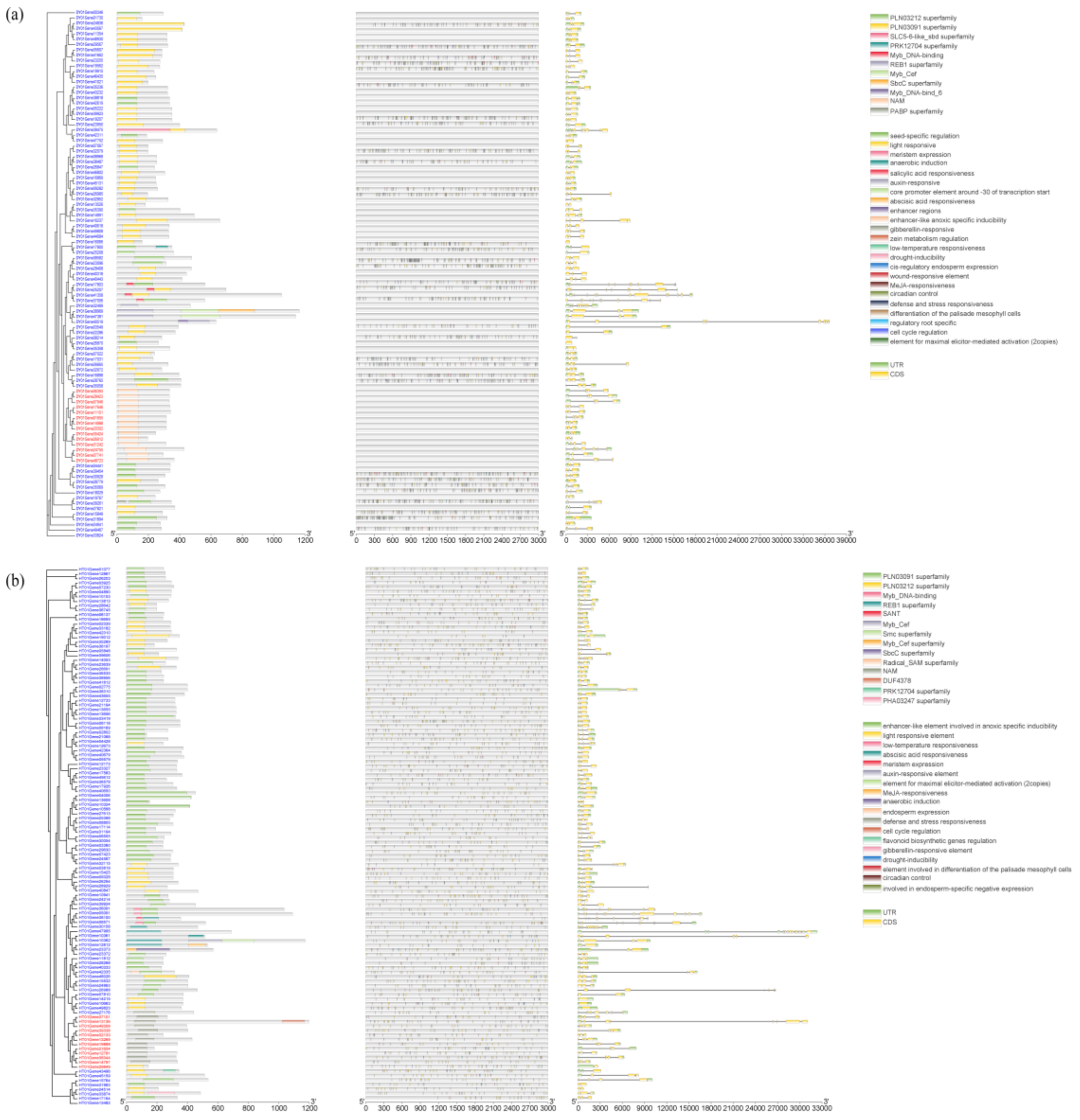

Fig. S4 Gene structure and prediction of the *cis*-regulatory elements and conserved domains of the *NACs* and *MYBs* in rattan. (a) *Calamus simplicifolius*. (b) *Daemonorops jenkinsiana*.

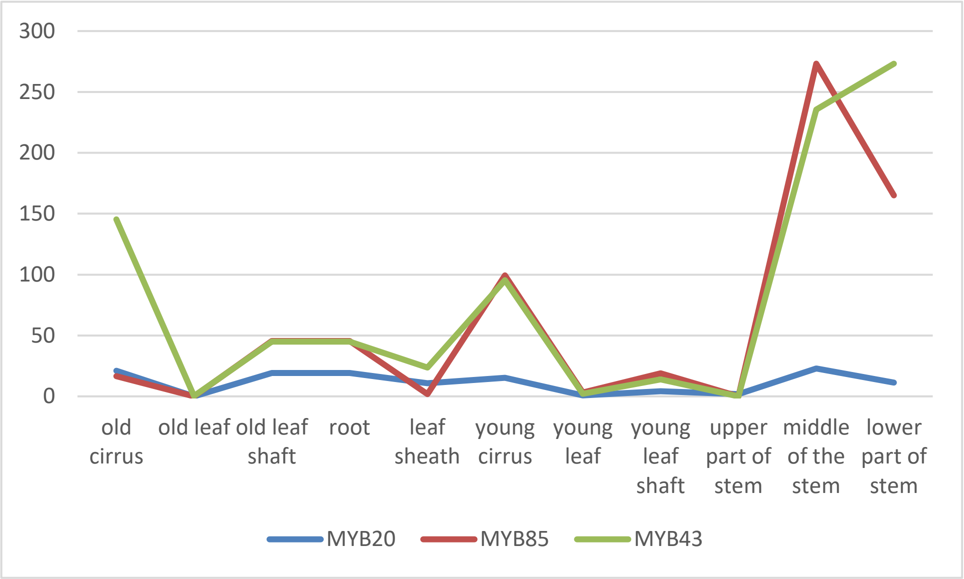

Fig. S5 Gene expression patterns of MYB20、MYB43、MYB85 in *Calamus simplicifolius.*

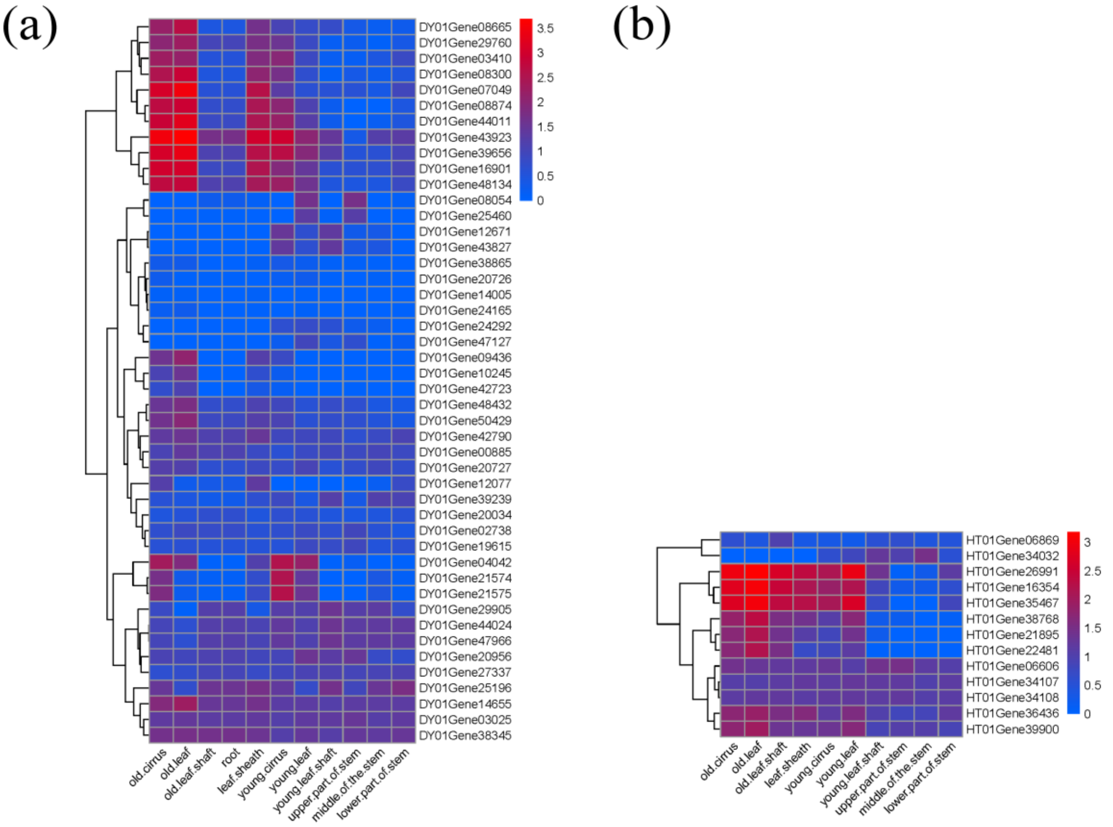

Fig. S6 Expression profile of the *LHC* gene family in rattan. (a) *Calamus simplicifolius.* (b) *Daemonorops jenkinsiana*.

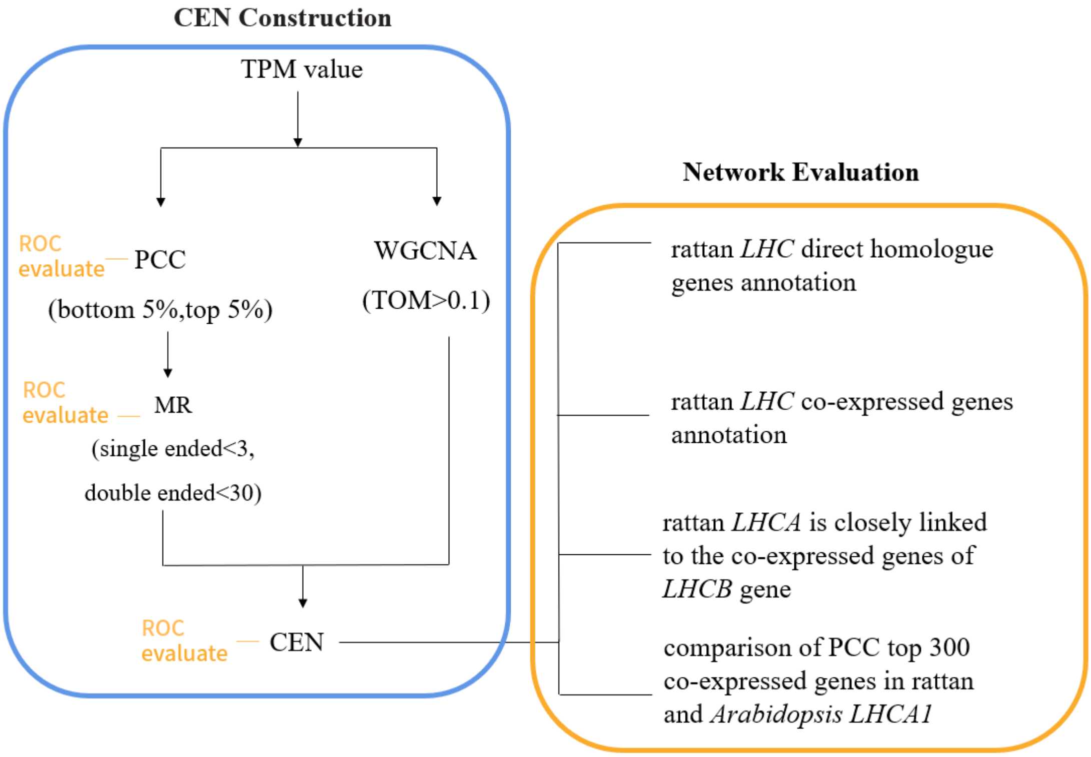

Fig. S7 Construction and evaluation of co-expression networks
